# Machine learning-assisted assessment of extracellular vesicles can monitor cellular rejection after heart transplant

**DOI:** 10.1038/s43856-025-00999-0

**Published:** 2025-07-11

**Authors:** Jacopo Burrello, Stefano Panella, Ilaria Barison, Chiara Castellani, Alessio Burrello, Lorenzo Airale, Jessica Goi, Veronica Dusi, Roberto Frigerio, Gino Gerosa, Chiara Tessari, Nicola Pradegan, Giuseppe Toscano, Giovanni Pedrazzini, Mattia Corianò, Francesco Tona, Sara Bolis, Alessandro Gori, Marina Cretich, Marny Fedrigo, Annalisa Angelini, Lucio Barile

**Affiliations:** 1 Cardiovascular Theranostics, Istituto Cardiocentro Ticino, Laboratories for Translational Research, Ente Ospedaliero Cantonale, Bellinzona, Switzerland; 2 Division of Internal Medicine and Hypertension Unit, Department of Medical Sciences, University of Torino, Torino, Italy; 3 Cardiovascular Pathology and Pathological Anatomy, Department of Cardiac, Thoracic, Vascular Sciences and Public Health, University of Padova, Padova, Italy; 4 Department of Electrical, Electronic and Information Engineering (DEI), University of Bologna, Bologna, Italy; 5 Division of Cardiology, Department of Medical Sciences, University of Torino, Torino, Italy; 6 National Research Council of Italy, Institute of Chemical Science and Technologies (SCITEC-CNR), Milan, Italy; 7 Division of Cardiac Surgery, Department of Cardiac, Thoracic, Vascular Sciences and Public Health, University of Padova, Padova, Italy; 8 Division of Cardiology, Istituto Cardiocentro Ticino, Ente Ospedaliero Cantonale Lugano Switzerland, Lugano, Switzerland; 9 Faculty of Biomedical Sciences, Università della Svizzera italiana, Lugano, Switzerland; 10 Cardiology Unit, Department of Cardiac, Thoracic, Vascular Sciences and Public Health, University of Padova, Padova, Italy; 11 Euler Institute, Faculty of Biomedical Sciences, Università Svizzera italiana, Lugano, Switzerland

**Keywords:** Predictive markers, Immunology

## Abstract

**Background:**

Heart transplant rejection, particularly acute cellular rejection (ACR), remains a critical post-operative concern, despite declining incidence rates. Current diagnostic standards rely on invasive endomyocardial biopsy, which presents limitations in sensitivity and reproducibility. There is an unmet need for noninvasive, accurate biomarkers that can detect and monitor rejection. This study aims to evaluate whether extracellular vesicle (EV) surface antigens, analyzed through flow cytometry and interpreted with artificial intelligence (AI), can serve as reliable biomarkers for ACR detection and monitoring in heart transplant recipients.

**Methods:**

We conducted a prospective longitudinal cohort study involving 24 heart transplant recipients over a median follow-up of 303 days. A total of 285 blood samples were analyzed for EV surface antigens exploiting two flow cytometry-based protocols. An adaptive AI model (random forest regressor) was developed to interpret EV antigen profiles, dynamically calibrating thresholds per patient.

**Results:**

Here we show that 14 EV surface antigens progressively increase with ACR severity. These changes are evident even before histological diagnosis. The AI model achieves an accuracy of 93.3% at leave-one-out testing (AUC 0.968), and 78.9% at validation in an independent cohort (AUC 0.832), with high specificity and negative predictive value. EV profiling outperforms conventional biochemical markers and provides anticipatory insight into rejection dynamics.

**Conclusions:**

EV profiling, enhanced by patient-specific AI modeling, offers a powerful noninvasive method for early detection and monitoring of ACR. This approach holds the potential to reduce reliance on biopsies and tailor immunosuppressive strategies more precisely.

## Plain language summary

Heart transplant patients are at risk of their body rejecting the new heart, which can lead to serious health problems. Current methods to detect rejection often rely on invasive procedures and may not catch the problem early enough. This study looked at whether tiny particles in the blood, called extracellular vesicles, could help detect rejection earlier and more accurately. Researchers analyzed blood samples from heart transplant patients and used artificial intelligence to interpret the data. They found that changes in these particles could signal rejection even before traditional tests did. This non-invasive method could help doctors treat rejection sooner, reduce the need for risky procedures, and improve long-term outcomes for transplant patients.

## Abstract (web summary)

Burrello et al. combine extracellular vesicle profiling with adaptive artificial intelligence to monitor cell rejection after heart transplant. Heart transplant rejection is detected with high accuracy, outperforming biopsy and anticipating rejection before histological signs, offering a promising noninvasive tool for timely intervention and personalized monitoring.

## Introduction

The incidence of heart transplant rejection has been decreasing steadily in recent years, with the 1-year risk dropping from 22% (2005–2009) to 11.8% (2010–2018) after hospital discharge^1^. These rates still rank among the highest in solid organ transplantation. Acute cellular rejection (ACR), occurring in roughly one-third of patients in the first year post-transplantation, is a major barrier to the long-term survival of cardiac allografts^2^, emphasizing the need for the development of sensitive and noninvasive biomarkers to ensure timely detection and intervention. The International Society for Heart and Lung Transplantation (ISHLT) advises routine surveillance through endomyocardial biopsy (EMB) with standardized histologic grading for ACR^3^. Despite this, there are challenges, including invasiveness and high interrater variability in grading rejection. Circulating biomarkers and molecular diagnostics have demonstrated relevant predictive value in monitoring rejection, with emerging data suggesting their potential in the identification of post-transplant complications. Genomic (cell-free DNA), transcriptomic (mRNA and microRNA profiling), and proteomic (protein expression quantitation) methodologies have been evaluated for diagnosing post-transplant outcomes, albeit with varying levels of evidence^4,5^. In most cases, prospective studies have not consistently confirmed the clinical utility of molecular markers in monitoring heart allograft recipients^6^. Circulating extracellular vesicles (EVs) have emerged as valuable noninvasive biomarkers for allograft rejection, with the potential to reduce the number of needed EMB procedures^7–9^. EV-based biomarkers have demonstrated the ability to diagnose rejection and its immunological variants, humoral and cellular rejection, with satisfactory sensitivity and specificity^8,9^. However, it’s important to note that these studies are retrospective and cross-sectional, introducing a risk of bias.

Confirmation in prospective longitudinal cohorts would be crucial. In the present study, we have advanced beyond our previous work by conducting a longitudinal prospective exploratory evaluation to further confirm and expand earlier findings. We enrolled 24 patients for longitudinal evaluations, spanning a median follow-up period of up to 1 year. At each visit concomitant to EMB, we obtained venous sampling resulting in a total of 285 plasma samples. Additionally, we implemented a technological shift in sample capture, transitioning from antibody-based methods to membrane-sensing peptides (MSPs)^10^, which facilitated the capture of circulating EV from complex biofluids. Finally, through supervised learning, we developed and internally validated a diagnostic model capable of dynamically adapting to specific patients to detect rejection episodes by profiling EV surface antigens. Our comprehensive approach blends prospective validation, advanced technological methodologies, and artificial intelligence (AI) to create a robust noninvasive diagnostic tool for monitoring heart transplant recipients.

## Materials and methods

### Study design and patient selection

**Fig. 1:**
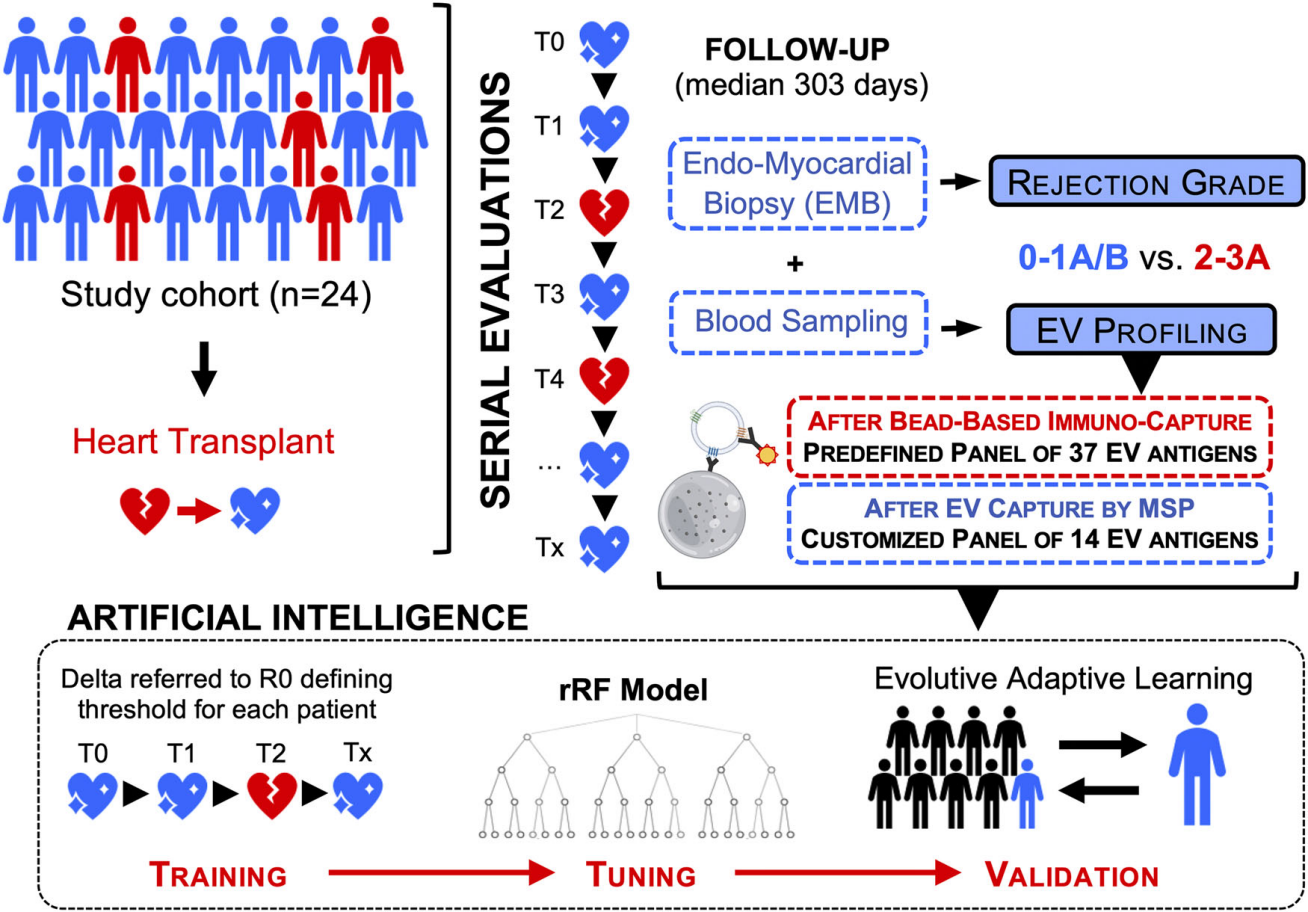
Study design. A total of 24 patients were enrolled and longitudinally evaluated for a median follow-up of 303 days, with a total number of visits ranging between 9 and 17 for each subject (total number of visits was 285). At each visit, patient underwent venous sampling and endomyocardial biopsy (EMB); a plasma sample was stored and used for EV profiling by flow cytometry (FC) according to two different protocols (see also methods): standardized immuno-capturing bead-based kit vs. customized EV profiling after capturing by membrane-sensing peptides (MSPs). From a predefined panel of 37 markers commonly expressed on EV membrane, we selected 14 EV antigens differentially expressed in rejecting, to be measured with our customized method (MSP-capturing). Levels of expression of EV antigens were combined in a biomolecular fingerprint by supervised learning, to build an AI random forest regressor (rRF) model and predict rejection episodes. Part of the figure was produced using the Servier Medical Art public domain (https://smart.servier.com).

Consecutive patients undergoing heart transplants between August 2020 and August 2021 were recruited and longitudinally evaluated for the first year after transplant. The study protocol (#0062556) was approved by the local ethical committee (University of Padova), and fully informed written consent was provided by each participant. A total of 24 patients were included in the analysis, with 9–17 visits each (visit median interval of 28 days); at each visit patient underwent clinical evaluation, routine biochemical exams, EMB, and blood sampling (blood was collected immediately before biopsy, thus avoiding confoundings related to the procedure). A total of 285 samples were collected and analyzed; the investigators who conducted experimental analysis were blind to patients’ diagnoses (Fig. 1).

Diagnosis and grading (from grade 0 to 3A) of ACR were defined according to guidelines of the ISHLT (see Supplementary Methods for the classification). Antibody-mediated rejection (AMR) was detected in two samples (patient #20), which were excluded from further analysis. To avoid confoundings due to systemic acute/chronic inflammation, samples from subjects with concomitant infections, active cancer, and/or autoimmune disease were excluded from the study.

### Characterization of EV surface antigens

**Fig. 2:**
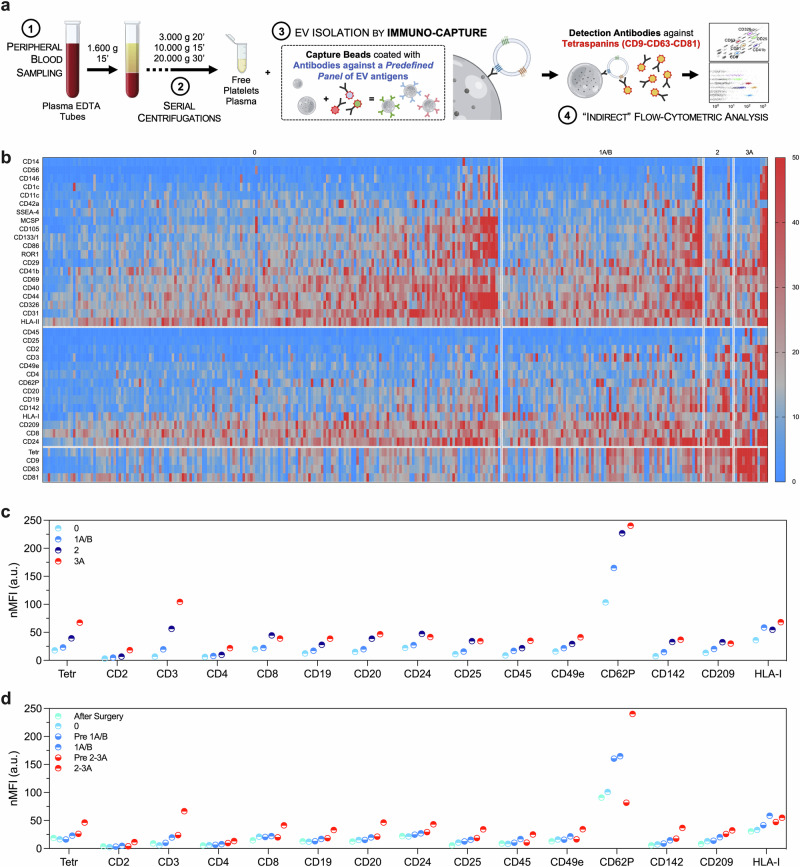
EV surface antigen profiling after immuno-capturing in rejecting recipients. Standardized profiling of surface antigens after EV isolation by immuno-capturing. Normalized median fluorescence intensity (nMFI; expressed as an arbitrary unit, a.u.) is reported for tetraspanins (CD9, CD63, and CD81) and for a predefined panel of 37 markers commonly expressed on EV membrane (285 samples included in the analysis). **a** Protocol for EV profiling after immuno-capturing. **b** Heat map showing EV surface antigen expression in patients stratified for rejection grade (from 0 to 3A; blue, low fluorescence; red, high fluorescence levels). **c**, **d** Median expression levels of EV surface antigens differentially expressed in rejecting patients, after patient stratification for time point of evaluation and rejection grade (*n* = 285): after surgery (first sampling after heart transplant); grade 0 (non-rejecting patients); ACR grade 1A/B; pre 1A/B (time point of evaluation before a diagnosis of ACR grade 1A/B); ACR grade 2–3A; pre 2–3A (time point of evaluation before a diagnosis of ACR grade 2–3A). Source data and statistics are reported in Supplementary Data S2 and S3. Part of (**a**) was produced using the Servier Medical Art public domain (https://smart.servier.com).

**Fig. 3:**
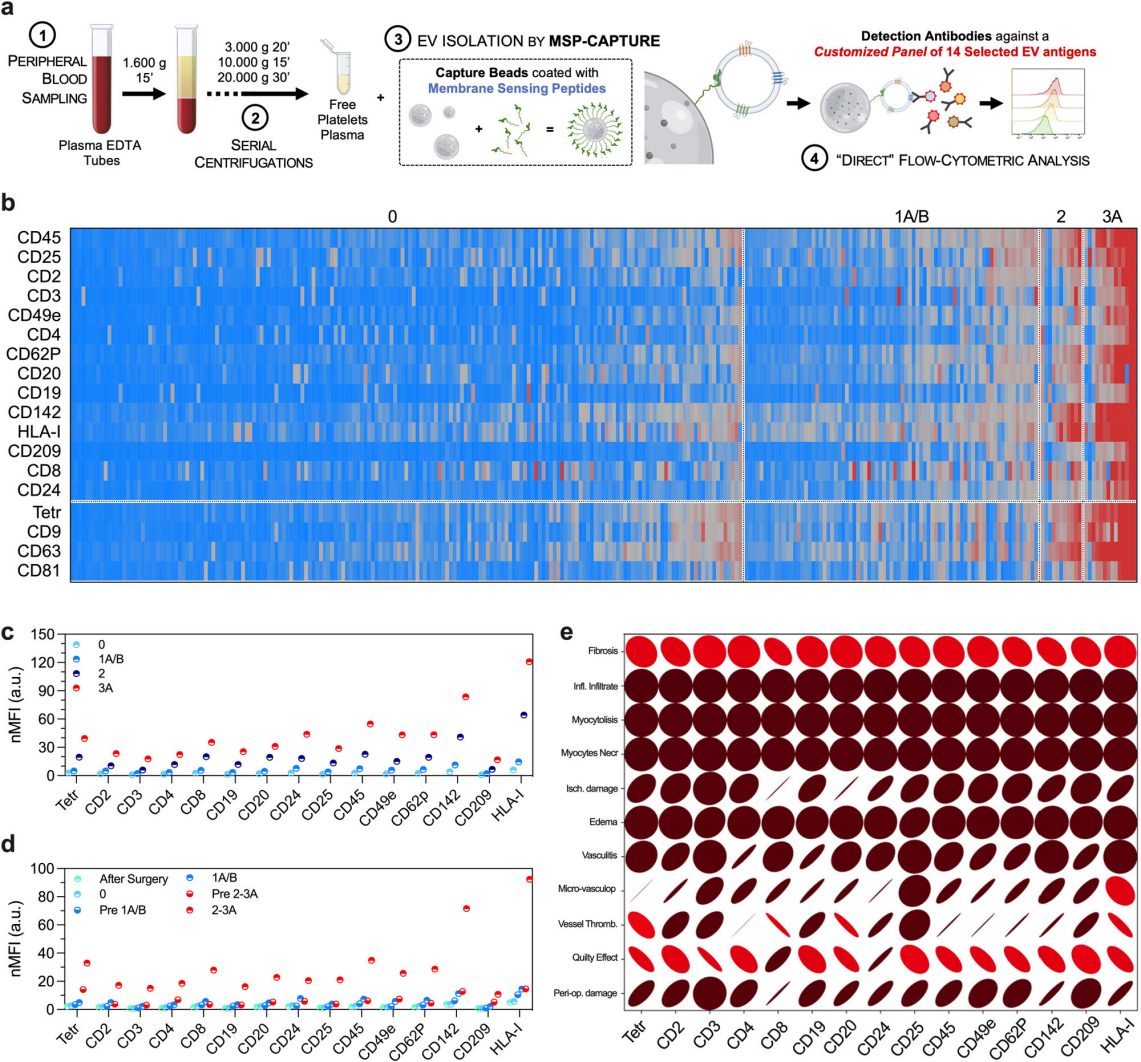
EV surface antigen profiling after MSP-capturing in rejecting recipients. Customized profiling of surface antigens after EV isolation by membrane-sensing peptide (MSP)-capturing. Normalized median fluorescence intensity (nMFI; expressed as an arbitrary unit, a.u.) is reported for tetraspanins (CD9, CD63, and CD81) and for a selected panel of 14 EV markers differentially expressed in rejecting recipients (*n* = 285). **a** Protocol for EV profiling after MSP-capturing. **b** Heat map showing EV surface antigen expression in patients stratified for rejection grade (from 0 to 3A; blue, low fluorescence; red, high fluorescence levels). **c**, **d** Median expression levels of EV surface antigens after patient stratification for time point of evaluation and rejection grade (*n* = 285): after surgery (first sampling after heart transplant); grade 0 (non-rejecting patients); ACR grade 1A/B; pre 1A/B (time point of evaluation before a diagnosis of ACR grade 1A/B); ACR grade 2–3A; pre 2–3A (time point of evaluation before a diagnosis of ACR grade 2–3A). **e** Association of single EV surface antigens with characteristics of endomyocardial biopsy; the slope indicates a direct or an inverse association, the ellipse radius the *P* value (a higher radius corresponds to a lower *P* value) while colors indicate the strength of the association (blue for an odds ratio ranging between 0 and 1, red for an OR from 1 to infinite). Source data and statistics are reported in Tables S2, S3 and S10. Part of (**a**) was produced using the Servier Medical Art public domain (https://smart.servier.com).

Blood was drawn into tubes containing EDTA and centrifuged at 1600 × *g* for 15 min to precipitate cellular components; low-centrifuge speed was used to prevent platelet activation due to shear-stress−induced. Free-platelet plasma underwent serial centrifugation cycles to remove cellular debris and larger EVs: 3000 × *g* 20 min, 10,000 × *g* 15 min, and 20,000 × *g* 30 min at 4 °C. All samples underwent systematic profiling of EV surface antigens according to two different protocols. A predefined panel of 37 antigens was evaluated by a standardized commercially available kit (Fig. 2a; MACSPlex Human Exosome Kit; Miltenyi Biotec, Bergisch Gladbach, Germany; named “EV profiling after immuno-capturing” throughout the manuscript)^11,12^. EV markers differentially expressed in rejecting patients were then included in an in-house customized panel to quantify EV antigens using flow cytometry after capture by MSP (named “EV profiling after MSP-capturing” throughout the manuscript; Fig. 3a; see Supplementary Methods and Table S1)^10^. EV antigen levels were expressed as median fluorescence intensity (MFI), normalized against the mean levels of tetraspanins CD9, CD63, and CD81 (normalized MFI, nMFI).

### Artificial intelligence, statistics, and reproducibility

IBM SPSS Statistics 26 (IBM Corp, Armonk, NY) and GraphPad Prism 9.0 (GraphPad, La Jolla, CA) were used for descriptive statistics. Parametric and non-parametric tests, Pearson’s *R* correlation test, and linear regression analyses were applied to assess associations between EV markers, biochemical routine exams, EMB parameters, and ACR diagnosis. The diagnostic performance of single EV markers was assessed by analysis of receiver operating characteristic (ROC) curves. A *P* value lower than 0.05 was considered significant.

AI (supervised learning algorithms) was applied to build a random forest regressor (rRF) model; Python 3.8.10 (library, scikit-learn 1.3.1) was used for model development. Supervised learning was exploited at two levels: (i) to formulate predictions on the primary endpoint (ACR grade 2–3A) on the base of measured levels of single EV markers expressed as nMFI after correction for median levels of each antigen in correspondence of grade 0 episodes for each single patient and reported as percentage of variation; see Supplementary Methods. (ii) To adapt thresholds of ACR detection to single patients during the respective follow-up; at each subsequent visit, the AI model considers only grade 0 episodes encountered at patient’s follow-up until the time point of evaluation, evolving and dynamically adapting to that specific patient, and continuously re-defining the threshold of variation associated with a high probability of rejection.

To correct for dataset imbalance, three different oversampling algorithms were applied to the dataset: synthetic minority oversampling technique (SMOTE), SMOTE and nearest neighbors, and random oversampling (RO). A grid search technique was applied to select the best oversampling algorithm and to tune the hyperparameters of the rRF. At validation, the most accurate rRF model was composed of 10 classification trees with a maximum number of splits equal to 10, corrected by the RO algorithm. The AI model was validated by a leave-one-out algorithm, which randomly selects *N*−1 patients, trains the rRF into this cohort and tests the trained model on the remaining subject; the process is reiterated *N* times (where *N* is the number of patients included in the analysis), with the test subject rotating at each round. Model accuracy at validation results from the mean of accuracy obtained at each round on the test patient.

### Reporting summary

Further information on research design is available in the Nature Portfolio Reporting Summary linked to this article.

## Results

### Characteristics of the study cohort

A prospective cohort of 24 consecutive patients, who underwent heart transplant in the same referral center, according to the same protocols, was enrolled and longitudinally followed with scheduled visits for a median of 303 days (from 9 to 17 visits each, with a median visit interval of 28 days); mean age was 58 years, and 83.3% were males. At each visit, recipients underwent clinical evaluation, routine biochemical exams, blood sampling, and EMB, with a total of 285 assessments (Fig. 1): 181 grade 0 and 104 episodes of ACR (28.1% grade 1A/B, 3.5% grade 2, and 4.9% grade 3A; Table 1). Graft trajectories are available in Fig. S1: grade 2–3A rejections occurred in 14 out of 24 enrolled patients, with 3 subjects experiencing 3 episodes (patients #10, #22, and #24). At the first visit after heart transplant, recipients were normotensive, with a mean BMI of 24.7 kg/m^2^, a mildly reduced renal function (mean eGFR 65 mL/min), and preserved left and right ventricular functions. Only one patient (4.2%) reported the presence of mild symptoms (dyspnea) with slight limitation during ordinary activity (NYHA class II; Table 1).

**Table 1:** Patients’ characteristics at baseline

Variable	Study cohort (*n* = 24)
Sex (ref. Male; *n*, %)	20 (83.3)
Age at transplant (years)	58 ± 12.5
Weight (kg)	74 ± 15.6
BMI (kg/m^2^)	24.7 ± 4.73
Systolic BP (mmHg)	119 ± 19.6
Diastolic BP (mmHg)	68 ± 12.4
Heart rate (apm)	90 ± 14.7
Creatinine (μMol/L)	121 ± 69.1
eGFR (mL/min)	65 ± 28.5
AST (U/L)	30 ± 14.7
ALT (U/L)	34 ± 27.7
LV ejection fraction (%)	63 ± 5.0
TAPSE (mm)	18 ± 3.4
sPAP (mmHg)	31 ± 8.9
Valvular disease^a^ (ref. Yes; *n*, %)	11 (54.2)
NYHA Class	
Class I (*n*, %)	23 (95.8)
Class II (*n*, %)	1(4.2)
Follow-up period (days)	303 [248; 362]
Follow-up visit interval (days)	28 [14; 35]
Rejection episodes	
Grade 0	181 (63.5)
Grade 1A/B	80 (28.1)
Grade 2	10 (3.5)
Grade 3A	14 (4.9)

Patients’ characteristics at baseline (*n* = 24; first visit after heart transplantation); the table also shows percentage and grade of rejection episodes at follow-up (overall number of evaluations = 285).

*BP* blood pressure, *eGFR* estimated glomerular filtration rate, *LV* left ventricular, *NYHA classification* New York Heart Association, *sPAP* systolic pulmonary artery pressure, *TAPSE* tricuspid annular plane systolic excursion.

^a^At least mild cardiac valvular disease. Parameters were indicated as mean ± standard deviation, median [and interquartile range], or absolute number (and percentage), as appropriate.

Biochemical parameters, immunosuppressive treatment, and characteristics of EMB at follow-up are summarized in Supplementary Data S1, after stratification for rejection grade. From clinical recordings at time points corresponding to an ACR grade 2–3A episode (*n* = 24), we observed that 91.7% of patients were asymptomatic, whereas one subject was symptomatic for dyspnea (NYHA class II), and another displayed a worsening of renal retention indices and of cardiac valvular function (mild-to-moderate mitral regurgitation and severe tricuspid regurgitation). At the biochemical evaluation, patients displayed higher levels of monocytes and basophils associated with 3A ACR, while no differences were found in other white blood cell populations, and indices related to liver and renal function. The immunosuppressive drugs used after heart transplantation (including cyclosporine, prednisone, mycophenolate, azathioprine, tacrolimus, and/or everolimus) were the same across the different rejection grades. As expected, according to histopathologic definition by current recommendations, inflammatory infiltrate, myocytolysis, myocyte necrosis, ischemic damage, edema, and vasculitis were observed more frequently in rejecting patients and specifically in correspondence with grade 2–3A ACR.

### Profiling of circulating EV surface antigens in heart transplant recipients

To pinpoint EV surface immune antigens that consistently reflect the ongoing cellular inflammatory process associated with ACR, we screened peripheral plasma samples obtained post-transplant and prior to each EMB at scheduled visits. EVs were characterized using an indirect flow cytometric approach. Plasma-derived vesicles were isolated with beads coated with antibodies targeting specific antigens of interest (37 distinct markers). The expression of these antigens was assessed in conjunction with the presence of tetraspanins, and pan-EV markers (CD9, CD63, and CD81), following a previously validated protocol (Fig. 2a)^11^. We identified 14 differentially expressed EV antigens (CD2, CD3, CD4, CD8, CD19, CD20, CD24, CD25, CD45, CD49e, CD62P, CD142, CD209, HLA-I): their median levels progressively increased in patients experiencing ACR from grade 1A/B to 3A (Fig. 2b, c; Supplementary Data S2). We independently validated the selected EV markers using a reverse methodological approach, involving the EV capture by a novel MSP designed to isolate small EVs^10,13^. MSP-captured EVs were directly stained with a tailored panel of labeled antibodies targeting tetraspanins and the 14 selected antigens (see methods; Fig. 3a). We confirmed that levels of expression of all the EV markers included in the customized panel gradually and significantly increased from non-rejecting patients to grade 3A (Fig. 3b, c; Table S2).

To further assess the predictive value of differentially expressed EV markers, we systematically categorized their expression levels at pre-determined time points. These time points included the first sampling after heart transplant (recognizing potential effects of surgery on EV subpopulations due to endothelial damage and inflammation), and samples collected at visits scheduled in correspondence with histological diagnosis, distinguishing between non-rejecting episodes (grade 0), grade 1A/B ACR, and grade 2–3A ACR. Grades 2 and 3A ACR were grouped together because they represent an indication to adjust immunosuppressive treatment, whereas 1 A/B ACR are usually considered as mild episodes and managed with a closer follow-up, even if there is no conclusive evidence^2,3^, and patient management is left to single-center experience. Additionally, we looked separately at time points before grade 1A/B ACR (pre 1A/B) and those before 2–3A ACR (pre 2–3A). This analysis demonstrated a distinct fingerprint of EVs obtained from samples collected at 2–3A episodes. Moreover, we observed that EV markers were already increased at time points prior to the histological detection of 2–3A rejection, as compared to non-rejecting patients (whose cardiac biopsies showed no histological signs of ACR); however, these markers were not increased above the levels detected during 1A/B episodes (Figs. 2d, 3d; Supplementary Data S3 and Table S3). Therefore, we compared EV markers after stratification of patients according to a low vs. high likelihood of moderate/severe grade rejection (Table S4; 0–1A/B vs. pre 2–3A vs. 2–3A episodes); a significant increase of CD2, CD24, CD49e, and CD62P was observed at pre 2–3A compared to 0–1A/B, thus suggesting that an EV signature may also serve as a potential early indicator of rejection, preceding histological assessment.

### Detection of ACR using normalized levels of single EV antigens after MSP-capturing

Given the interindividual variability observed in circulating EV subpopulations^14^, we opted for a patient-specific normalization approach to enhance the diagnostic accuracy of our EV signature. This approach involved dividing the absolute expression of each EV marker by the respective median levels recorded during non-rejecting episodes (grade 0) for that specific marker and patient: the resulting values were then considered as a percentage increase or decrease compared to grade 0, facilitating the establishment of reliable cut-offs applicable to a broader cohort.

As expected, normalized levels of EV surface antigens after MSP-capturing displayed significant increases at ACR grade 1A/B, 2, and 3A diagnosis as compared to grade 0 (G0-delta percentage variation ranging between +201 and +314% for grade 1/AB, between +576 and +1094% for grade 2, and between +1788 and 4577% for grade 3A; Table S5). In addition, we observed an increase in EV antigen normalized levels also in correspondence of pre 1A/B episodes (from +4.3% to +131%) and pre 2–3A (from +82% to +539%; Table S6). Expression levels of EV marker after normalization for grade 0 levels and for every single patient are reported in Fig. S2.

Univariate regression analysis confirmed the association of all the evaluated EV antigens with ACR (grade 2–3A diagnosis). Delta variation values displayed OR ranging between 1.035 for CD3 and CD8 to 1.369 for CD142, thus meaning an increase from 3.5 to 36.9% in the likelihood of ACR grade 2–3A for each 1% increase in median levels of that specific EV antigen as compared to grade 0 levels for that patient (*p* < 0.001; Table S7).

The analysis of ROC curves allowed us to identify cut-offs for the diagnosis of grade 2–3A ACR using absolute values and normalized levels of each EV antigen; the accuracy was generally very high for all the evaluated markers, with an AUC ranging between 0.785 and 0.937 for normalized EV delta variation levels (*p* < 0.001; Table S8), and sensitivity and specificity up to 100 and 98.5%, respectively. As an example, a variation equal to or higher than +429.2% as compared to patient-specific G0 median levels for CD45 displayed a sensitivity/specificity of 83.3/86.6% in the diagnosis of ACR.

### EV surface signature and clinical correlations

Direct weak correlations were found between several EV antigens (levels obtained after MSP-capturing), levels of basophils and renal function (Pearson’s R coefficients ranging between 0.124 and 0.313; Table S9); interestingly, we did not observe any other significant correlation between antigens carried by EVs (and differentially expressed in rejecting patients), and the evaluated biochemical parameters, thus suggesting that EV signature, but not the conventional biochemical profile, changed during rejection. On the other side, all the evaluated EV markers were associated with EMB features related to ACR, including inflammatory infiltrate, myocytolysis, myocyte necrosis, and edema (OR ranging between 1.01 and 1.16; Fig. 3e and Table S10).

Of note, a multivariate regression model was built to assess the association between single EV antigens and the identification of ACR, after correction for immunosuppressive treatment (cyclosporine-based vs. tacrolimus-based regimen). All EV antigens differentially expressed in rejecting patients were associated with grade 2–3A ACR, independently from the assumed therapy (Table S11). After diagnosis of grade 2 or 3A ACR, immunosuppressive regimen was adjusted according to current guidelines; consistently, the expression of all EV antigens associated with ACR (except CD8) significantly decreased after treatment variation (Table S12), mirroring the clinical improvement of patients (see also single patients’ graft trajectories; Figs. S1 and S2).

### Development and validation of an AI model to predict heart rejection

**Fig. 4:**
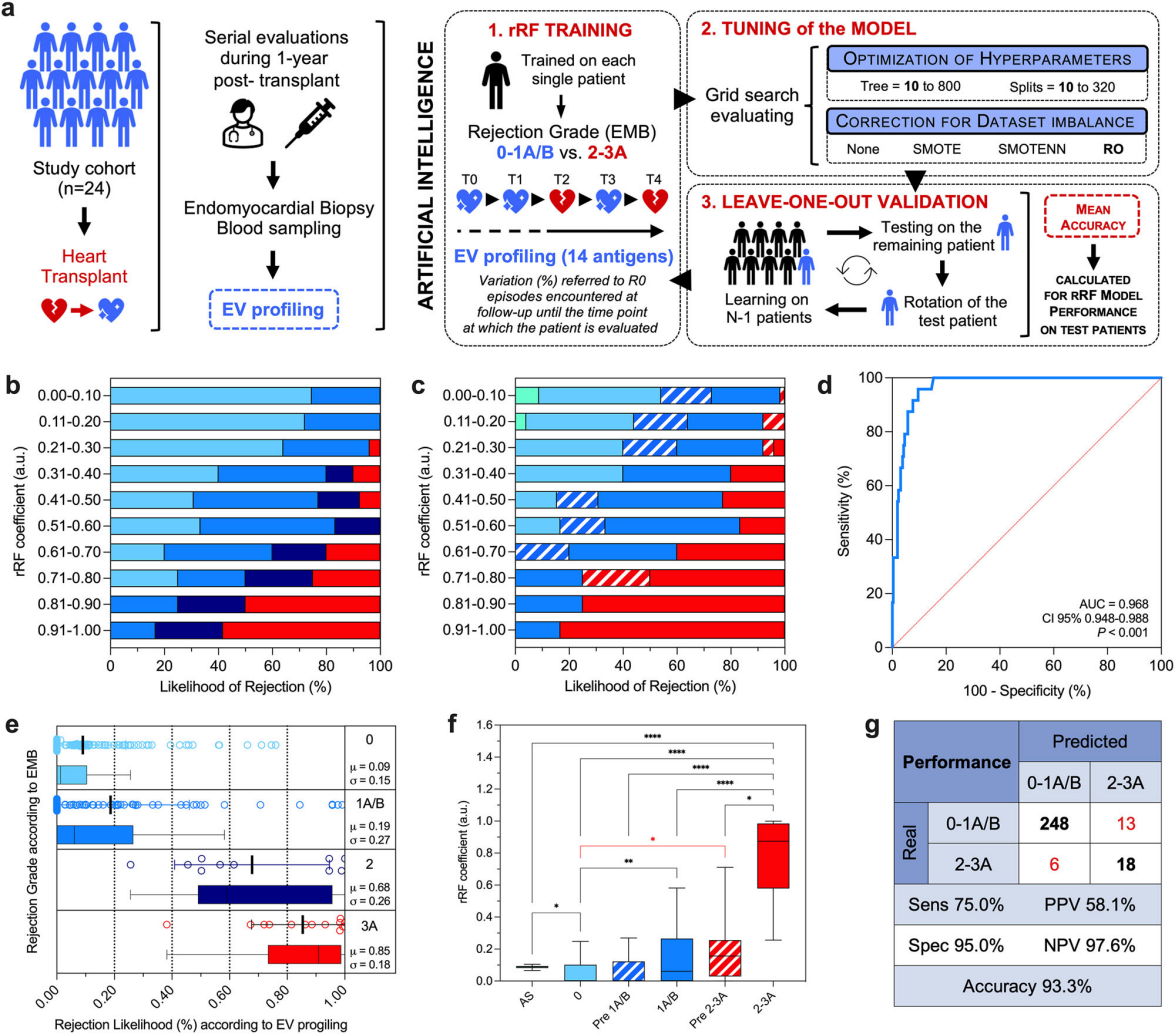
AI model to longitudinally predict rejection episodes. Supervised learning was used to build a diagnostic predictive model based on artificial intelligence to detect rejection episodes exploiting EV surface antigen profiling performed after membrane-sensing peptide (MSP)-capturing (285 samples included in the analysis). **a** A random forest regressor (rRF) model was trained, tuned, and validated through a leave-one-patient-out strategy (see methods). The rRF model combines levels of expression of EV antigens in a biomolecular fingerprint, based on normalized fluorescence intensity after correction for median levels of each antigen in correspondence with non-rejecting episodes (G0) for each single patient, and reported as a percentage of variation. The AI model considers, at each subsequent visit, only the G0 episodes encountered during the patient’s follow-up until the time point at which the patient is evaluated, evolving, and dynamically adapting to that specific patient, and continuously re-defining the threshold of variation associated with a high probability of rejection. **b**, **c** Likelihood of rejection after stratification of patients for rRF coefficients. **d** ROC curve analysis; area under the curve (AUC) together with 95% confidence interval (CI) is reported for rRF coefficient discriminating ACR grade 2–3A from G0-1A/B episodes. **e** Median values of rRF, distribution, and likelihood of rejection of patient stratified according to rejection grade (from 0 to 3A; *n* = 285). **f** Box plot and interquartile range for rRF coefficients in patients stratified according to the time point of evaluation and rejection grade (*n* = 285): after surgery (first sampling after heart transplant); grade 0 (non-rejecting patients); ACR grade 1A/B; pre 1A/B (time point of evaluation before a diagnosis of ACR grade 1A/B); ACR grade 2–3A; pre 2–3A (time point of evaluation before a diagnosis of ACR grade 2–3A). **g** Diagnostic performance (sensitivity, specificity, accuracy, positive and negative predictive values) at rRF model validation. Source data and statistics are reported in Supplementary Data S4 and Table S13. Part of (**a**) was produced using the Servier Medical Art public domain (https://smart.servier.com).

AI was applied to develop a model based on supervised learning algorithms. This model can identify ACR by analyzing unique EV fingerprints associated with grade 2–3A diagnosis. This fingerprint is generated by combining the expression levels of specific EV antigens that are differentially expressed during rejection as compared to grade 0 within the same patient. AI was used to process delta-normalized values obtained at each subsequent visit: it considers only grade 0 episodes encountered at patient’s follow-up until the time point of evaluation, continuously and dynamically re-defining the threshold of variation (increase/decrease from G0 levels) associated with a high probability of rejection for that specific patient (Fig. 1). To this goal, we used an rRF model; rRF was built to discriminate ACR grade 2–3A from 0 to 1A/B diagnosis, using the 14 EV antigens differentially expressed in rejecting patients and measured by FC after MSP-capturing. Training of the AI model, tuning, and validation strategies are described in the methods section (Supplementary Data S4 and Fig. 4a).

The rRF model generates a coefficient corresponding to the probability of ACR grade 2–3A for the considered sample; increasing coefficients were directly correlated with the proportion of subjects with rejection and may be used to define the likelihood of rejection for each analyzed sample: 9 ± 15% non-rejecting patients (grade 0); 19 ± 27% for grade 1 A/B; 68 ± 26% for grade 2; 85 ± 18% for grade 3A (Fig. 4b–e and Table S13). The analysis of the ROC curve demonstrated a reliable performance with an AUC of 0.968 (95% CI 0.948–0.988; Fig. 4d) and an accuracy of 93.3% at validation: 248 of 261 G0-1A/B episodes were correctly classified resulting in a specificity of 95%, and 18 of 24 ACR grade 2–3A were detected with a sensitivity of 75% (Fig. 4g); positive and negative predictive values were 31.9% and 96.2%, respectively. The net benefit of the AI model compared to the use of single EV antigens (see ROC curve analysis for delta variations in Table S8) was estimated by comparing their performance in discriminating ACR grade 2–3A: the AI model applied to EV profiles after MSP-capturing displayed a 15% and 9.9% increase, respectively in mean accuracy and AUC.

The profiling of circulating EVs collected at the visits scheduled before a grade 2–3A diagnosis at EMB still resulted in a significantly higher risk of ACR compared to G0 samples (Fig. 4c–f; Table S13); moreover, median rRF coefficients at time points before a diagnosis of 2–3A ACR were higher, even if not significantly, compared to ACR 1A/B (Fig. 4f). Consistently, the unsupervised clustering of EV profiles after MSP-capturing (regardless EMB) allowed a clear discrimination of the majority of samples according to their final diagnosis (Fig. S3).

**Fig. 5:**
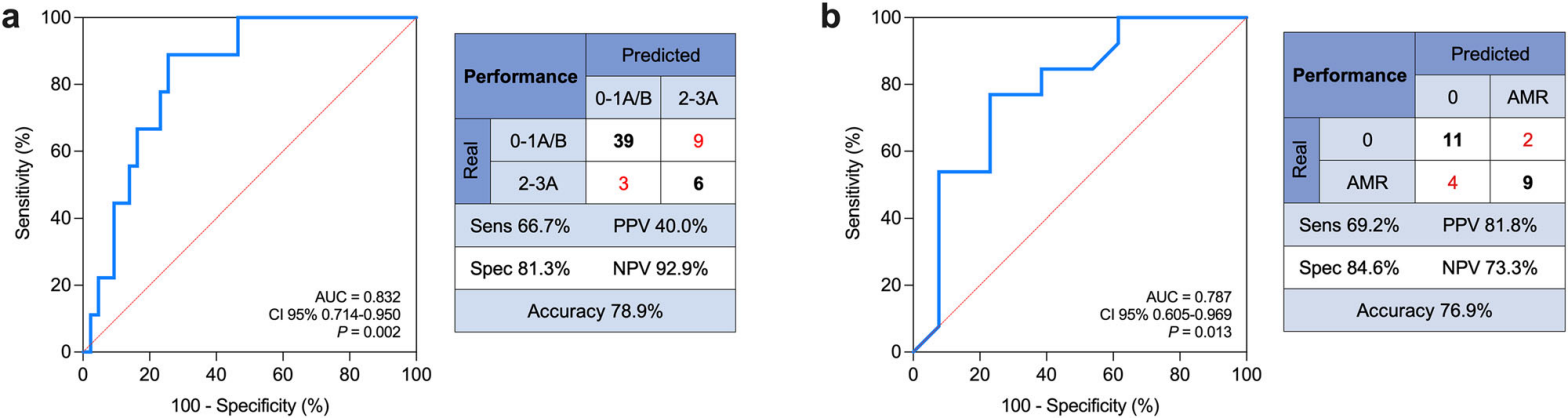
Validation of the AI model applied to EV profiling after MSP-capturing. The AI model was tested on **a** an independent cohort of patients (*n* = 5) followed up to 357 days (57 samples), and **b** a separate cohort of subjects with a diagnosis of antibody-mediated rejection (AMR; *n* = 13). Diagnostic performance (sensitivity, specificity, accuracy, positive and negative predictive value) are reported together with ROC curve analysis (area under the curve, AUC, with 95% confidence interval) and *P* value for the comparison with referral line. Source data and statistics are reported in Tables S14 and S15.

The AI model was then validated in an internal independent validation cohort of patients (*n* = 5; Fig. 5 and Table S14), with a follow-up of up to 347 days (57 evaluated samples). The accuracy was 78.9%, with a specificity of 81.3%, a negative predictive value of 92.9%, and an AUC of 0.832 at analysis of the ROC curve (Fig. 5a).

**Fig. 6:**
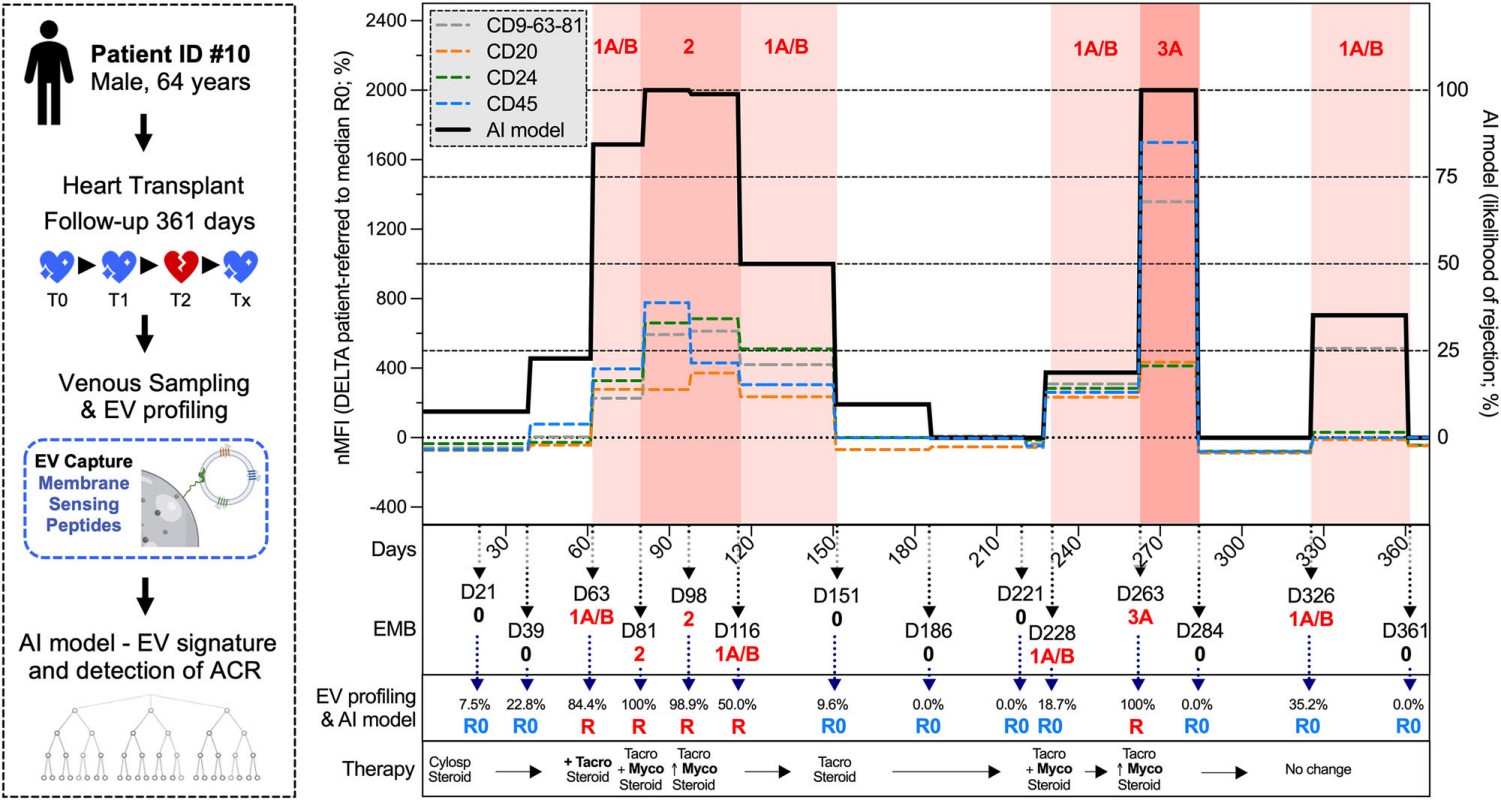
Application of AI to monitor cellular rejection after heart transplant. Artificial intelligence (AI) and the profiling of circulating extracellular vesicles (EVs) by MSP (membrane-sensing peptide) -capture are applied to monitor patient #10, a 64-year-old male who underwent a heart transplant and was followed for 361 days after the heart transplant. Days (D) after transplant, result of the endomyocardial biopsy (EMB), and immunosuppressive therapy are reported for each evaluated time point, together with levels of EV surface antigens (tetraspanins, CD20, CD24, and CD45, are showed as exemplary representation) and output of the AI model (likelihood of ACR grade 2–3A according to the EV signature, basing on normalized levels of EV antigens with dynamic adaptation of decisional thresholds for this specific patient; see methods). Part of the figure was produced using the Servier Medical Art public domain (https://smart.servier.com).

The reliability of our EV marker is exemplified by individual patients’ data (Figs. 6 and S2). The EV signature dynamically changes in response to rejection episodes. While EV markers, such as tetraspanins, CD20, CD24, and CD45 increased during the initial episode of ACR grade 1A/B after 63 days, and during subsequent episodes of ACR grade 2 and 3A occurring after 81 and 263 days, respectively, their expression levels decreased following changes in therapy. This decline reflects the diminished systemic inflammation resulting from immunosuppressive treatment. The AI model accurately predicted ACR grade 2–3A and two out of four grade 1A/B diagnoses. Additionally, using EV profiling, our AI model can estimate the likelihood of rejection at each evaluated time point; in this way, we observed an increased probability of rejection before the occurrence of ACR (e.g., increase from 7.5% to 22.8% at day 39, preceding ACR episodes at days 63 and 81). The theoretical application of our model may lead to select patients with an increasing probability of rejection at follow-up, for a closer re-evaluation, thus allowing a potentially earlier detection of ACR.

EMB and blood sampling corresponding to episodes of AMR were excluded from our analysis. Nevertheless, even if not trained for this scope, we tested our AI model on EV profiling of 13 patients with AMR from a previous study^9^ (Table S15). Of the 14 EV antigens differentially expressed in ACR, CD2-CD8-CD19-CD20-CD25-CD45-HLA-I were increased also in patients with a diagnosis of AMR compared to their baseline levels. The AI model correctly discriminated 76.8% of samples, with a specificity and negative predictive value of 84.6 and 73.3%, respectively, and an AUC of 0.787 (Fig. 5b).

As a technical assessment and to evaluate the reproducibility of EV profiling in the detection of ACR, the AI model was applied to median fluorescence levels obtained by EV profiling after antibody-mediated capturing, showing similar performance; rRF was able to effectively stratify most subjects according to their likelihood of ACR (Fig. S4a–e and Table S16), with a still reliable accuracy (AUC 0.854; accuracy of 85.6%, sensitivity/specificity of 62.5/87.7%, and positive/negative predictive value of 31.9/96.2%; Fig. S4f). The analysis of Bland-Altman plots confirmed a relatively low inter-assay variability (Table S17 and Fig. S5), with a 3% overestimation in tetraspanins MFI levels of MSP-capturing approach over immuno-capturing (quadratic and linear *R* coefficients of 0.561 and 0.555—*p* < 0.001, respectively, at correlation analyses; Fig. S5c); a 6.2% mean overestimation was observed when considering nMFI for all the other EV antigens.

The final refining of our AI model included the evaluation of its accuracy at the increase of the number of G0 episodes encountered in the graft trajectory of every single patient (from 85.4% for 2–5 G0, to 98.6% for 10–14 G0 episodes; Fig. S6a). The overfitting effect was relatively low (2.9 and 7% for MSP-capturing and immuno-capturing, respectively; Fig. S6b). The application of oversampling strategy resulted in an increase of sensitivity with a slight decrease in specificity, maintaining a comparable accuracy (after MSP-capturing: from 52.4/97.9/94.4% to 75.0/95.0/93.3% of sensitivity/ specificity/accuracy without and with oversampling, respectively; after immuno-capturing: from 8.3/98.9/91.2% to 62.5/87.7/85.6% of sensitivity/specificity/accuracy without and with oversampling; Fig. S6c), and the transfer learning applied to the two protocols resulted in a still acceptable performance (91.1% accuracy when the model was trained on EV profiling obtained by MSP-capturing and validated on data from immuno-capturing, and 82.8% with the reverse approach; Fig. S6d). A comparison between the accuracy of our AI model applied to EV profiling and the already available standard of care exploiting AlloMap and characterization of double-strand cell-free DNA, demonstrated superior performance and comparable performance, respectively (Table S18).

## Discussion

The key findings of this exploratory study are as follows: (i) The levels of specific EV-associated surface markers show a progressive increase from non-rejecting patients (grade 0) to ACR grade 3A diagnosis, offering a comprehensive understanding of the evolving dynamics of rejection. In contrast, traditional biochemical profiling struggles to differentiate rejection episodes, revealing only minor differences, mainly in monocytes and basophils, in correspondence with grade 3A episodes. (ii) The predictive capacity of EV markers at time points preceding the EMB identification of an ACR grade 2–3A suggests their potential for anticipating histological detection of rejection. (iii) We implemented an adaptive AI model that dynamically adjusts to each patient, normalizing EV antigens fluorescence levels and continuously refining cut-offs to discern rejection probabilities, thus overcoming the intrinsic variability of EV profiling due to patient characteristics, instrumental settings, and potential batch effects.

This innovation addresses two critical aspects: it first overcomes potential biases introduced by using data from different instruments, protocols, and settings; secondly, it mitigates relevant risks associated with treatment delays or unnecessary interventions, stemming from the limitations of histologic grading in predicting a patient’s clinical course. Through the utilization of a longitudinal approach, the AI model provides a nuanced “personalized” understanding of the evolving rejection process, rather than a simple binary “yes/no” answer. This is a valuable insight into rejection dynamics, potentially reducing delays in intervention or untailored variation of immunosuppressive therapy, by mirroring the evolution of rejection trajectories and treatment responses. This is exemplified by the clear dynamic correspondence observed in single patient data for EV surface antigen profiling. The normalized levels of expression of EV surface antigens exhibited substantial increases during rejection episodes (ACR grade 1A/B, 2, and 3A) compared to the baseline (G0). Conversely, when immunosuppressant therapy was introduced or adjusted, the level of expression decreased accordingly, reflecting clinical improvement of patients and graft trajectories.

The diagnostic and predictive value of EVs lies in their ability to promptly reflect the dynamic changes occurring in the intricate cellular processes associated with ACR. The orchestration of graft tolerance and rejection involves various immune cells. Initially, antigen-presenting cells (APCs) work with CD4+ helper cells to activate CD8+ cytotoxic T cells, which migrate to the graft. Target cell recognition is facilitated by MHC class I molecules, leading to apoptosis induced by perforin and granzyme B, along with TNF-α secretion. Allo-specific CD4+ T cells, activated by APCs, release pro-inflammatory factors like IL-1, IFN-γ, and TNF-α. These factors attract monocytes, macrophages, and eosinophils, which produce harmful agents causing graft damage. By profiling circulating EV, the active role of APCs is highlighted by the presence of dendritic cell-specific intercellular adhesion molecule (CD209) vesicles, and the progressive involvement of cytotoxic T cells is mirrored by a gradual increase in the level of expression of specific markers such as CD2 and CD3. The progressive involvement of cytotoxic T cells is confirmed by the predominant correlation of conventional T-cell markers with grade classification^15^. In our study this is evident by the gradual increase in the expression levels of specific markers such as CD8, CD2, and CD3. CD3 and CD8 have been identified as effective diagnostic markers in ROC curve analysis, aligning with findings from immunohistochemical counting of inflammatory cells in EMB by Bocchi et al., which demonstrated a robust correlation between cellular rejection grade and CD3+ cell counts^16^. Furthermore, patients exhibiting poor responses to treatment in ACR grade 2 displayed higher CD3+ cell counts^16^. This was recently confirmed by proof-of-concept studies applying a state-of-the-art, fully quantitative, multiplex immunofluorescence methodology showing that high-grade EMB exhibit significantly elevated levels of CD3+ and CD8+ cells compared to low-grade EMB^17^. Similarly, regardless of grade classification, severe, clinically evident rejection events show higher proportions of CD3+ and CD8+ cells compared to clinically silent rejection events^17^. All these findings taken together provide further insights toward a tailored patient-centered approach, and even if beyond the scope of the present study, the change in EV patterns after ACR and as a response to an adjustment of immunosuppressive regimen may suggest new therapeutic options, including circulating EVs as potential targets.

It is noteworthy that while the utility of detecting conventional markers at the histological level in EMB^17^ and on circulating cells (as evidenced by the absence of significant correlation between lymphocytes and grade of rejection in our study) is limited in distinguishing between cases with and without serious clinical rejection syndromes, these same markers associated with circulating EV effectively discriminate between ACR grade 2–3A and G0. Indeed, the ability of EV to capture changes in surface antigen expression of activated cells adds a layer of precision to rejection monitoring that conventional biochemical methods fail to achieve. Moreover, the high negative predictive value observed for our AI model highlights, as a potential application, the identification of non-rejecting patients to be deferred over time, reducing the number of potentially unnecessary EMB. Overall, by overcoming the limitations of histologic grading, which include the lack of long-term risk stratification and the difficulty in customizing surveillance testing and immunosuppression-weaning protocols to individual patients’ ACR risk profiles, EV profiling has the potential to assist clinicians in optimizing patient care, minimizing unnecessary interventions, and enhancing overall outcomes in heart transplant recipients. Moreover, the dynamic shift in EV antigens measured with our assay from grade 1 to 3A episodes, when compared to G0, underscores the potential of these markers in detecting also milder forms of rejection, enabling closer patient monitoring.

On the other side, we were unable to perform a patient-matched, side-by-side comparison of our EV-based method with established screening methods for rejection, such as gene expression profiling and donor-derived cell-free DNA. However, when comparing performance with previous studies, our AI model demonstrated effectiveness comparable to that of cell-free DNA-based techniques in identifying ACR^18–20^, while showing slightly superior performance compared to the results reported in the literature for gene expression profiling^21^. These approaches have become accepted standardized screening methods for allograft heart rejection.

Another notable strength of the model lies in its technical validation through two distinct approaches. The system was tested with a change in capture technology, moving from antibody-based methods to utilizing sensing peptides^10^, alongside modifications in the analysis workflow. With the use of immuno-capturing beads, each conjugated with specific antibodies, different subpopulations of EV were enriched, but this approach lacked an exhaustive view of the circulating vesicles. In contrast, employing MSP-coated beads facilitated the pan-capturing of the entire population of circulating EVs, which were then stained for specific markers. This method primarily relied on the frequency of these EV-marker pairs rather than on their expression levels within specific subpopulations, which could be influenced by the number of captured EVs. Remarkably, the model demonstrated high specificity and accuracy in both scenarios, underscoring its generalizability across different technical approaches and instruments. The adopted normalization approach ensures that each patient’s response is assessed relative to their own baseline. This reduces the influence of inter-patient variability and minimizes potential biases caused by confounding factors; additionally, it addresses the limitations associated with longitudinal evaluations involving repeated measures on a restricted number of individuals. With regard to clinical translatability of our model, the turnaround time from sampling to data analysis ranges between 12 and 14 h down to 5 h according to the applied protocols^22^, with a coefficient of variability between 5.8 and 20.7%, from previous studies validating the immuno-capture approach using alternative protocols, instruments, type, and amount of biological sample^11,23,24^.

The exclusion of AMR represents the main limitation of the study: AMR is a distinct form of rejection with its own pathophysiology. The exclusion of AMR from the analysis may hamper the full translation of our AI model in clinical practice, thus failing to provide a comprehensive understanding of this type of rejection; anyway, the retrospective application of AI to EV profiling from a previous study^9^ demonstrated an acceptable diagnostic performance, suggesting the feasibility of future studies dedicated to the identification of AMR. A second limitation of our study is the lack of external validation. While we employed a leave-one-out validation approach and further validated the AI model using an independent internal cohort of patients, including those with AMR, validation using data from external cohorts and/or laboratories would enhance the robustness and generalizability of our findings. Such validation would provide deeper insights into the impact of immunosuppressive regimens beyond cyclosporine and tacrolimus, as well as inter-laboratory variability in measurements. Moreover, patients with concomitant infections, active cancer, and/or autoimmune disease were excluded, thus hampering the possibility to assess if the diagnostic reliability would be preserved in these categories of patients. Finally, even if the employment of a predefined panel of EV surface antigens represents a standardizable approach and is easily translatable to clinical practice, it may have missed potentially relevant biomarkers that could have been assessed by an unbiased proteomic approach.

In conclusion, we demonstrated that EV profiling may represent a noninvasive tool to monitor recipients of heart transplants; we provided evidence of consistent performances in detecting allograft rejection through a leave-one-out validation approach and at internal validation in independent cohorts of patients displaying ACR or AMR, with accuracies up to 93.3, 78.9, and 76.9%, respectively. Technical replicability was assessed using different protocols for EV isolation and characterization (MSP- and immuno-capturing) and by application of transfer learning. Our AI model could be integrated into a user-friendly interface requiring input of measured fluorescence levels for single EV antigens, with the rejection likelihood as output: patients with a higher probability of rejection may be selected for a closer follow-up, while in case of low probability, the follow-up will be delayed. This will allow an early and tailored treatment, potentially increasing the survival of cardiac allografts. The application of AI, which dynamically adapts to individual patients and refines thresholds for identifying rejection with each new assessment, demonstrates strong utility in identifying non-rejecting patients. While the positive predictive value of our AI model is relatively low, its ability to effectively rule out rejection enhances its potential for precision medicine, supporting more targeted and individualized patient management.

## Supplementary information

Supplemental Information

Description of Additional Supplementary Files

Supplementary Data S1

Supplementary Data S2

Supplementary Data S3

Supplementary Data S4

Reporting summary

## Data Availability

All data needed to evaluate the conclusions in the paper are present in the paper and/or the Supplementary Materials. Additional information may be obtained from corresponding authors upon reasonable request. Source data for Fig. 2 is in Supplementary Data S2 and S3; source data for Fig. 3 is in Supplementary Tables S2, S3 and S10; source data for Fig. 4 is in Supplementary Data S4 and Table S13; source data for Fig. 5 is in Supplementary Tables S14 and S15.

## Abbreviations

ACR: acute cellular rejection
AI: artificial intelligence
AMR: antibody-mediated rejection
AUC: area under the curve
EMB: endomyocardial biopsy
EV(s): extracellular vesicle(s)
FC: flow cytometry
ISHLT: International Society for Heart and Lung Transplantation
MFI: median fluorescence intensity
MSPs: membrane-sensing peptides
ROC: receiver operating characteristic
rRF: random forest regressor

## Code availability

The underlying code for this study is publicly available on GitHub at the following link: https://github.com/ABurrello/EV-HT^25^.
