# Supplementary material for: Machine learning-assisted assessment of extracellular vesicles can monitor cellular rejection after heart transplant
Source: Commun Med (Lond). 2025 Jul 11;5:288. doi: 10.1038/s43856-025-00999-0 (PMC12246144; doi:10.1038/s43856-025-00999-0)
Supplement: Supplementary file 1 — Supplemental Information [file 43856_2025_999_MOESM1_ESM.pdf]

ONLINE SUPPLEMENTARY INFORMATION

Machine learning-assisted assessment of extracellular vesicles can monitor cellular rejection after heart transplant

Jacopo Burrello[1,2*], Stefano Panella[1*], Ilaria Barison[1,3*], Chiara Castellani[3], Alessio Burrello[4], Lorenzo Airale[2], Jessica Goi[2], Veronica Dusi[5], Roberto Frigerio[6], Gino Gerosa[7], Chiara Tessari[7], Nicola Pradegan[7], Giuseppe Toscano[7], Giovanni Pedrazzini[8,9], Mattia Corianò[10], Francesco Tona[10], Sara Bolis[1], Alessandro Gori[6], Marina Cretich[6], Marny Fedrigo[3], Annalisa Angelini[3#], Lucio Barile[1,9#].

Affiliations: [1]Cardiovascular Theranostics, Istituto Cardiocentro Ticino, Laboratories for Translational Research, Ente Ospedaliero Cantonale; Bellinzona, Switzerland. [2]Division of Internal Medicine and Hypertension Unit, Department of Medical Sciences, University of Torino, Torino, Italy. [3]Cardiovascular Pathology and Pathological Anatomy, Department of Cardiac, Thoracic, Vascular Sciences and Public Health, University of Padova, Padova, Italy. [4]Department of Electrical, Electronic and Information Engineering (DEI), University of Bologna, Bologna, Italy. [5]Division of Cardiology, Department of Medical Sciences, University of Torino, Torino, Italy. [6]National Research Council of Italy, Institute of Chemical Science and Technologies (SCITEC-CNR), Milan, Italy. [7]Division of Cardiac Surgery, Department of Cardiac, Thoracic, Vascular Sciences and Public Health, University of Padova, Padova, Italy. [8] Division of Cardiology, Istituto Cardiocentro Ticino, Ente Ospedaliero Cantonale Lugano Switzerland, Lugano, Switzerland. [9]Faculty of Biomedical Sciences, Università della Svizzera italiana, Lugano, Switzerland. [10]Cardiology Unit, Department of Cardiac, Thoracic, Vascular Sciences and Public Health, University of Padova, Padova, Italy. * These authors contributed equally; # Corresponding authors.

List of Contents

Supplementary Methods

Figure S1 – Graft trajectories of patients included for the development of the AI model
Figure S2 – Single patient longitudinal analysis
Figure S3 – Unsupervised clustering of EV profiles after MSP-capturing
Figure S4 – Validation of the AI model with EV profiling after bead-based immune-capture
Figure S5 – Bland-Altman plot of tetraspanins detected after MSP- vs. immuno-capturing
Figure S6 – AI to detect rejection episodes: performance at tuning of the model

Table S1 – Protocols for EV isolation and surface antigens characterization
Table S2 – EV surface profiling after MSP-capturing (14 antigens) according to rejection episodes
Table S3 – EV surface profiling after MSP-capturing according to time-point of evaluation
Table S4 – EV surface profiling after MSP-capturing: sub-analysis on rejection episodes
Table S5 – Normalized levels of EV surface antigens after MSP-capturing during rejection episodes
Table S6 – Normalized levels of EV surface antigens after MSP-capturing at time-point analysis
Table S7 – Association of single EV surface antigens with the diagnosis of rejection
Table S8 – Diagnostic performance of single EV surface antigens
Table S9 – Correlation analysis of EV profiling and biochemical patients' profile
Table S10 – Association analysis of EV profiling and endomyocardial biopsy characteristics
Table S11 – Association of EV markers and rejection independently from immunosuppressive therapy
Table S12 – EV surface profiling after MSP-capturing: sub-analysis on immunosuppressive treatment
Table S13 – Likelihood of rejection according to AI and EV profiling after MSP-capturing
Table S14 – EV surface profiling after MSP-capturing in an independent validation cohort
Table S15 – EV surface profiling after immuno-capturing in antibody-mediated rejection
Table S16 – Likelihood of rejection according to AI and EV profiling after immuno-capturing
Table S17 – Bland-Altman analysis of EV profiling after MSP- vs. immuno-capturing
Table S18 – Diagnostic performance of AI model and EV profiling compared to literature

Suppl. Data S1 – Clinical-biochemical parameters and endomyocardial biopsy during rejection episodes
Suppl. Data S2 – EV surface profiling after immuno-capturing (standardized panel of 37 antigens)
Suppl. Data S3 – EV surface profiling after immuno-capturing according to time-point of evaluation
Suppl. Data S4 – Training and Tuning of an AI model to predict rejection episodes

Supplementary References

Extended Methods

Study design and patient selection

Consecutive patients undergoing heart transplant between August 2020 and August 2021 at the Cardio-Surgery Center Gallucci (Department of Cardiac-Thoracic-Vascular Sciences, and Public Health at the University Hospital of Padua, Italy) were recruited and longitudinally evaluated for the first year after transplant. Study protocol (#0062556) was approved by local ethical committee and fully informed written consent was provided by each participant.

A total of 24 patients were included in the analysis, with 9-17 visits each (visit median interval of 28 days); at each visit patient underwent clinical evaluation, routine biochemical exams, EMB and blood sampling (blood was collected immediately before biopsy, thus avoiding confoundings related to the procedure). A plasma aliquot for each blood sample was centralized at the Laboratories for Translational Research - Cardiovascular Theranostics (Istituto Cardiocentro Ticino, Ente Ospedaliero Cantonale, Bellinzona, Switzerland) for EV profiling. A total of 285 samples were collected and analyzed; the investigators who conducted experimental analysis were blind to patients' diagnosis.

Diagnosis and grading (from grade 0 to 3A) of acute cellular rejection (ACR) were defined according to guidelines of the International Society for Heart and Lung Transplantation (1); Formalin-fixed paraffin-embedded bioptic samples were stained with H&E and immuno- histochemistry was performed on tissue sections with monoclonal antibodies anti-C4d (Biomedica Groupe, Vienna, Austria), and anti-CD68 (ClonePG-M1; Dako Cytomation, Milano, Italy) to identify AMR (2); EMB were carefully reviewed by two independent pathologists, with divergences solved by discussion. Antibody mediated rejection (AMR) was detected in two samples (patient #20), which were excluded from further analysis. According to clinical practice in our transplantation center, ACR grade 2 and 3A were grouped for analysis purpose. This strategy allowed the discrimination of patients at higher risk of major adverse events (3), who are treated specifically, from those who do not require changes of immunosuppression regimen (4); the coupling of ACR grade 2 with 3A and of samples from non-rejecting patients with ACR grade 1A/B was defined *a priori*, regardless EV profiling; the unsupervised clustering of EV profiles after MSP-capturing regardless patient diagnosis was consistent with this association as shown by the principal component analysis in Figure S3, thus excluding potential classification bias in our analysis.

Based on the mean estimated variability of normalized fluorescence intensity levels for the 37 evaluated EV antigens after immuno-capturing (see below), the inclusion of 24 patients and 285 samples (24 episodes of rejection grade 2-3A) corresponds to an estimate study power of 81.3% (Cohen coefficient of 0.623; α-error = 0.05).

Characterization of EV surface antigens

All samples underwent to systematic profiling of EV surface antigens according to two different protocols. A pre-defined panel of 37 antigens was evaluated by a standardized commercially available kit (Figure 2A; MACSPlex Human Exosome Kit; Miltenyi Biotec, Bergisch Gladbach, Germany), as previously described (named "EV profiling after immuno-capturing" throughout the manuscript) (5). After serial centrifugation steps, a 60 uL aliquot of pre-cleared plasma was used for bead-based, multiplex EV immuno-capture; briefly, plasma was diluted to a final volume of 120 uL with MACSPlex buffer (MPB) and incubated overnight (10-12h) on an orbital shaker (800 rpm at 10°C, protected from light) with MACSPlex Exosome Capture Beads, containing 37 antibody-coated differentially stained bead subsets (labelled with different amounts of phycoerythrin-[PE] and fluorescein isothiocyanate-[FITC]); MPB was used as a blank control. After incubation and two washing steps with MPB, 15 uL of MACSPlex Exosome Detection Reagent (5 uL for each allophycocyanin [APC]-conjugated anti-CD9, -CD63, and -CD81 detection antibody) were added and incubated for 60 minutes 450 rpm at 10°C, protected from light. After another washing step, samples were loaded onto and analyzed by MACSQuant Analyzer 10 flow cytometer (Miltenyi Biotec; Bergisch Gladbach, Germany). Median fluorescence intensity (MFI) was measured for each subset of capture beads, corrected by subtracting the signal of corresponding blank controls, and normalized by the mean MFI of CD9, CD63, and CD81 (normalized

MFI, nMFI). Multiplex platform analyses and gating strategies were previously described (6,7). Total turnaround time from sample collection to data analysis is 12-14 hours.
The 14 EV markers differentially expressed in rejecting patients were then included in an in-house customized panel to quantify EV antigens using flow cytometry after capture by MSP (named "EV profiling after MSP-capturing" throughout the manuscript; Figure 3A). Two uL of membrane sensing peptide-conjugated beads (capture beads) (8,9) were added to 15 uL of plasma samples with the volume was adjusted to 100 uL with PBS. Samples were incubated under 900 rpm agitation for 2 hours at room temperature. After the incubation, a magnetic separation step was performed to isolate the beads-EVs complexes. EV-bounds beads were subsequently split into two different tubes and incubated in the dark under 900 rpm agitation for 1 hour at room temperature, with specific staining mixture: in the first tube, we profiled the EV surface with an antibody cocktail composed of CD2-PE-Dazzle 594, CD4-BV421, CD8-PC7, CD9-BV510, CD19-BV650, CD25-AF700, CD49e-BV750, CD63-BV605, CD209-FITC, HLA-ABC APC (BioLegend, USA); CD3-BUV496, CD45-BUV615, CD62p-BUV805, CD81-BUV737 (BD Bioscience, USA); CD24-PE, CD142-PerCP Vio700 (Miltenyi Biotech, Germany); CD20-APC AF750 (Beckman&Coulter, USA). All the antibodies were used at a final concentration of 0.5 ug/mL, adjusting the staining volume to 100 uL with PBS supplemented with 0.5 mg/mL of human IgG and 0.5% human albumin, to reduce unspecific binding. In the second tube, we used the membrane dye Memglow700 (Cytoskeleton, USA) to normalize the membrane signal; MemGlow was used at a final concentration of 50 nM in 100 uL PBS. After staining, samples were magnetically separated to remove unbound dyes, resuspended in 100 uL of PBS, and acquired by Symphony A5 (BD Bioscience). At least 10'000 single-bead events were collected and further analyzed based on the median fluorescence in each specific channel. Turnaround time from sample collection to data analysis is approximately 5 hours. A summary of main steps of the adopted protocols for EV isolation and surface antigens characterization is reported in Table S1.

Statistical analysis
IBM SPSS Statistics 26 (IBM Corp, Armonk, NY) and GraphPad Prism 9.0 (GraphPad, La Jolla, CA) were used for statistics. Categorical variables were expressed as absolute number and percentage, and compared with chi-square test (or Fisher's test when appropriated). Scalar variables were analyzed by Kolmogorov-Smirnov test to evaluate the distribution: normally distributed variables were expressed as mean ± standard deviation and analyzed by ANOVA one-way with post-hoc Bonferroni's tests; non-normally distributed variables were expressed as median and interquartile range, and analyzed by Kruskal−Wallis test. Pearson's R test was applied to assess correlations between EV markers and biochemical routine exams. Linear regression models were used to evaluate associations between EV markers, EMB parameters and ACR diagnosis. Diagnostic performance of single EV markers and of artificial intelligence (AI) -models was assessed by receiver operating characteristics (ROC) curves and evaluation of the area under the curve (AUC); cut-offs corresponding to the highest accuracy were identified by Youden Index (J = sensitivity + specificity - 1). Analysis of Bland-Altman plots was performed after data transformation by Z-score (subtraction of the mean from the data point, and division of the result by standard deviation). A *P*-value lower than 0.05 was considered significant.

Artificial Intelligence
A rRF algorithm was built using a supervised learning method with Python 3.8.10 (library, scikit-learn 1.3.1). Supervised learning is applied to formulate predictions on the primary endpoint (ACR grade 2-3A) on the base of a pre-selected set of multi-dimensional paired input data (measured levels of single EV markers expressed as nMFI after correction for median levels of each antigen in correspondence of grade 0 episodes for each single patient and reported as percentage of variation; i.e., for patient #1, we calculated median florescence intensity of CD2 for grade 0 and then normalized each CD2 measurement for median $CD2_{G0}$-ID#1 according to the following equation: Delta-CD2ID#1 [%] = [(CD2ID#1)-(Median$CD2_{G0}$-ID#1)]/[(Median$CD2_{G0}$-ID#1+)]*[100)]). At each subsequent visit, the AI model considers only grade 0 episodes encountered at patient's follow-up until the time point of evaluation,

evolving and dynamically adapting to that specific patient, and continuously re-defining the threshold of variation associated with a significant probability of rejection.
The rRF algorithm creates a set of "n" classification trees with a maximum number of splits for each tree. The predicted endpoint results from the outcome of each classification tree of the forest; if at least (n/2) + 1 of "n" trees of the rRF predicts 2-3A rejection as outcome, then this endpoint is assigned to the sample. To correct for dataset imbalance (24 diagnosis of rejection grade 2-3A out of 285 analyzed samples; 8.4%) and avoid an accuracy paradox (falsely higher accuracy due to over-prediction of the most represented outcome), three different oversampling algorithms were applied to the dataset: synthetic minority over-sampling technique (SMOTE), SMOTE and nearest neighbors (SMOTENN), and random oversampling (RO). This approach imputes new simulated patient data starting from real patients, to balance the number of rejection occurrences with non-rejecting episodes.
A grid search technique was applied to select the best oversampling algorithm and to tune hyper-parameters of the rRF: i) number of classification trees (from 10 to 800), and ii) maximum number of splits (from 10 to 120). At validation, the most accurate rRF model was composed by 10 classification trees with a maximum number of splits equal to 10, corrected by RO algorithm.
The AI model was validated by a leave-one-out algorithm, which randomly selects N-1 patients, trains the rRF into this cohort and tests the trained model on the remaining subject; the process is reiterated N times (where N is the number of patients included in the analysis), with the test subject rotating at each round. Model accuracy at validation results from the mean of accuracy obtained at each round on the test patient.

Figure S1 – Graft trajectories of patients included for the development of the AI model

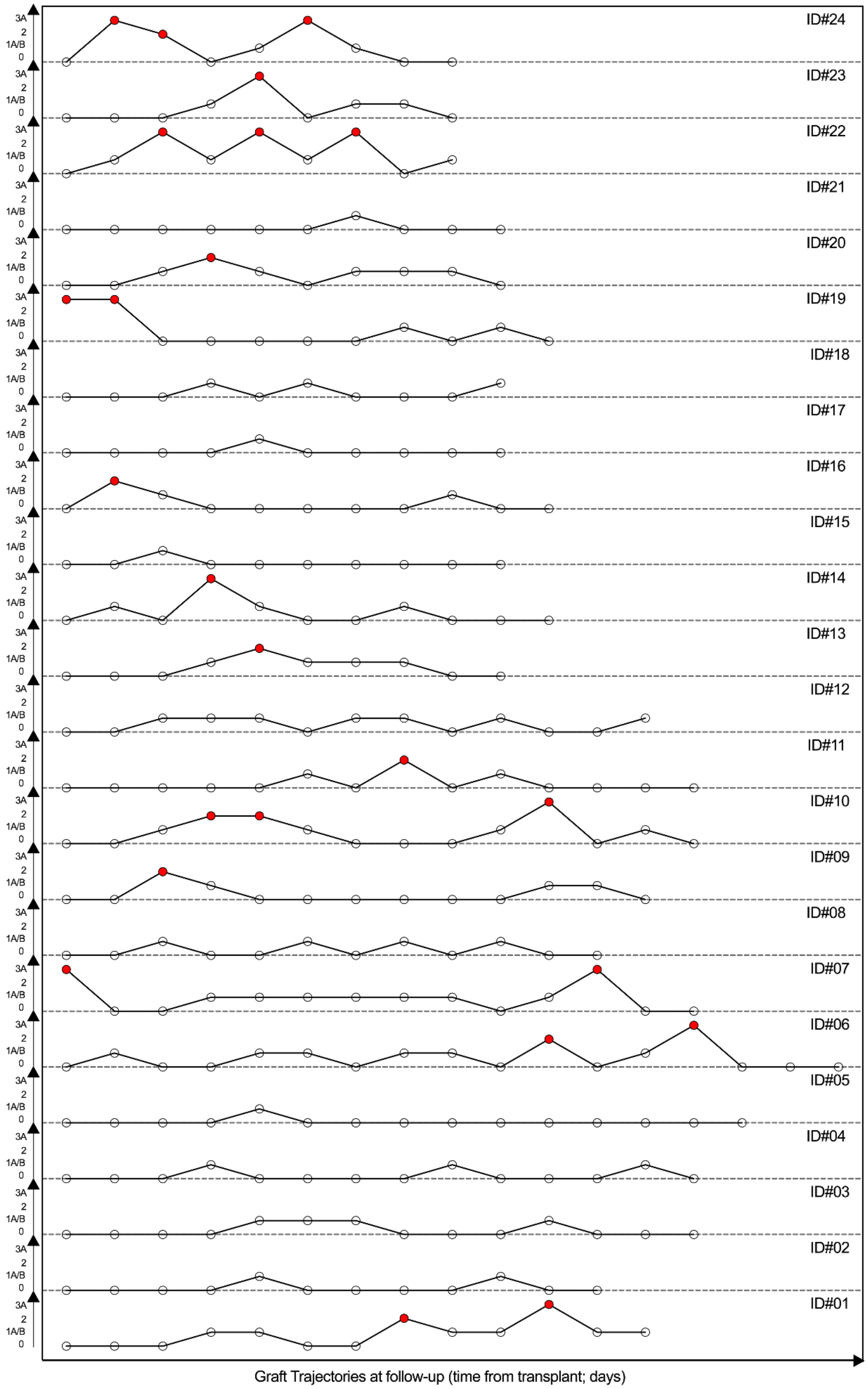

The panel shows graft trajectories of the 24 patients included in AI model development (red dots indicates diagnosis of rejection grade 2-3A).

Figure S2 – Single patient longitudinal analysis

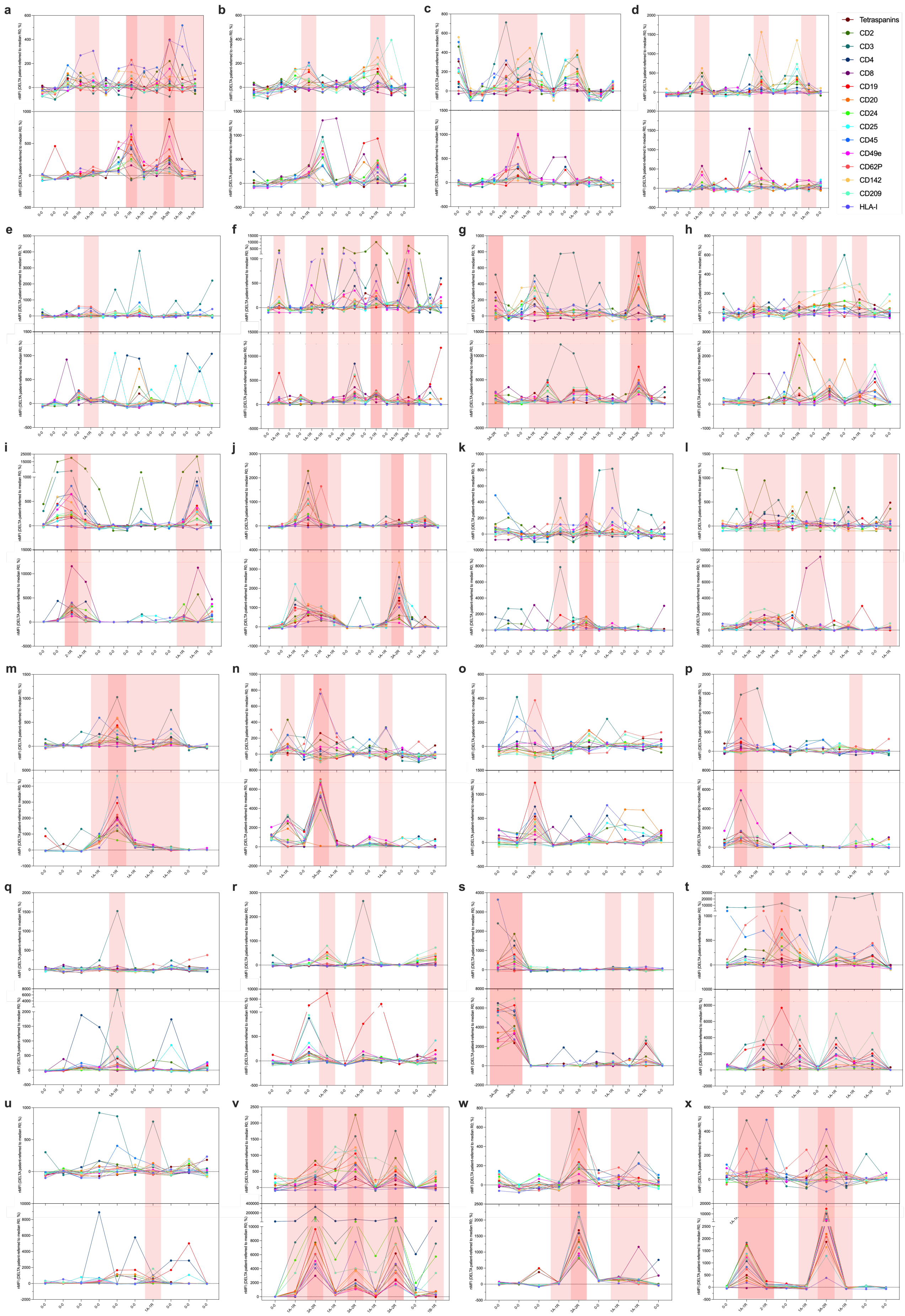

Single patient data for EV surface antigen profiling by standardized bead-based immuno-capturing (above) or by flow cytometry after EV isolation by membrane sensing peptides (MSP)- capturing (below). Data are expressed as normalized median fluorescence intensity (nMFI; %) after correction for median levels of each antigen in correspondence of grade 0 episodes for each single patient, and reported as percentage of variation (i.e., for patient #1, we calculated median MFI of CD2 for grade 0 and then normalized each CD2 measurement for median $CD2_{G0\text{-}ID\#1}$ according to following equation: *Normalized Delta-*$CD2_{ID\#1}$ *[%] = [(*$CD2_{ID\#1}$ *[a.u.])-(Median*$CD2_{G0\text{-}ID\#1}$ *[a.u.])]/ (Median*$CD2_{G0\text{-}ID\#1}$ *[a.u.])*100*).

Figure S3 – Unsupervised clustering of EV profiles after MSP-capturing

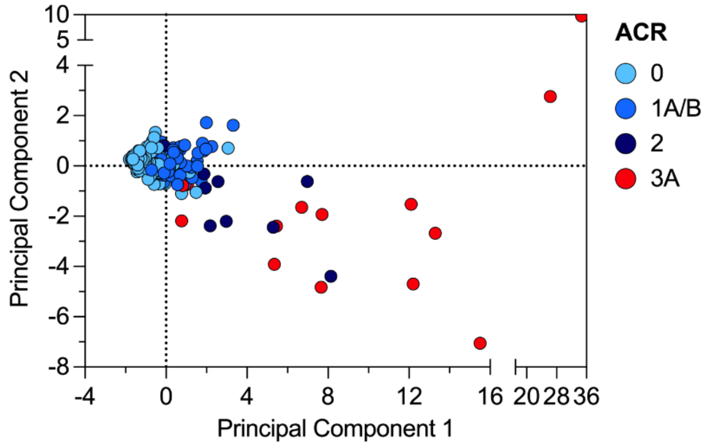

The plot shows the principal component analysis (PCA) of EV profiles after MSP-capturing regardless the diagnosis at EMB (n=285). Each point represents the EV profile (combination of the 14 surface antigens from the customized panel) of one analyzed sample; diagnosis are assigned after clusterization and reported by colors (grade 0, non-rejecting episodes; grade 1A/B acute cellular rejection, ACR; grade 2 ACR; grade 3A ACR).

Figure S4 – Validation of the AI model with EV profiling after bead-based immune-capture

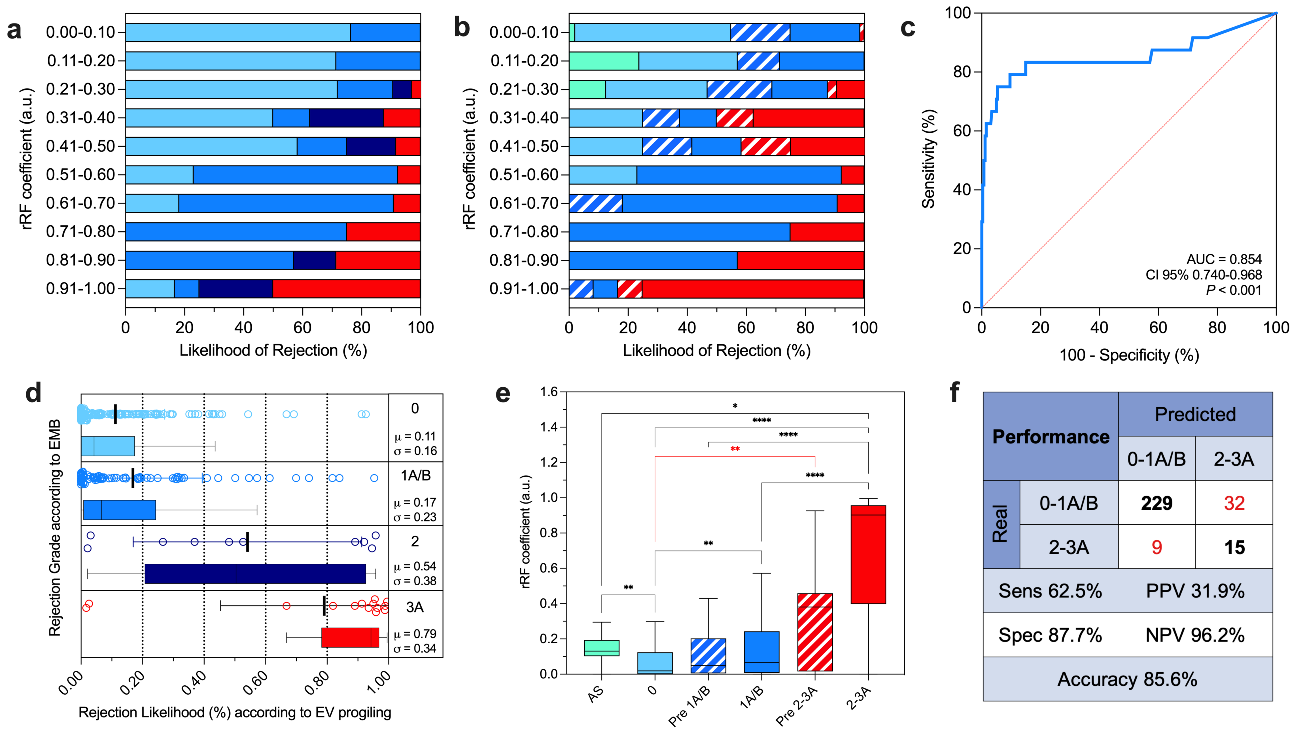

Supervised learning was used to build a diagnostic model based on artificial intelligence to detect rejection exploiting EV surface antigen profiling performed after immuno-capturing. (**a-b**) Likelihood of rejection after stratification of patient for rRF coefficients. (**c**) ROC curve analysis; area under the curve (AUC) together with 95% confidence interval (CI) is reported for rRF coefficient discriminating rejection grade 2-3A from 0-1A/B. (**d**) Median values of rRF, distribution and likelihood of rejection of patient stratified according to rejection grade (from 0 to 3A). (**e**) Box plot and interquartile range for rRF coefficients in patients stratified according to the time-point of evaluation and rejection grade: after surgery (first sampling after heart transplant); grade 0 (non-rejecting patients); grade 1A/B rejection; pre 1A/B (time-point of evaluation before a 1A/B diagnosis); grade 2-3A rejection; pre 2-3A (time-point of evaluation before a 2-3A diagnosis). (**f**) Diagnostic performance (sensitivity, specificity, accuracy, positive and negative predictive values) at rRF model validation. Source data and statistics are reported in Table S16.

Figure S5 – Bland-Altman plot of tetraspanins detected after MSP- vs. immuno-capturing

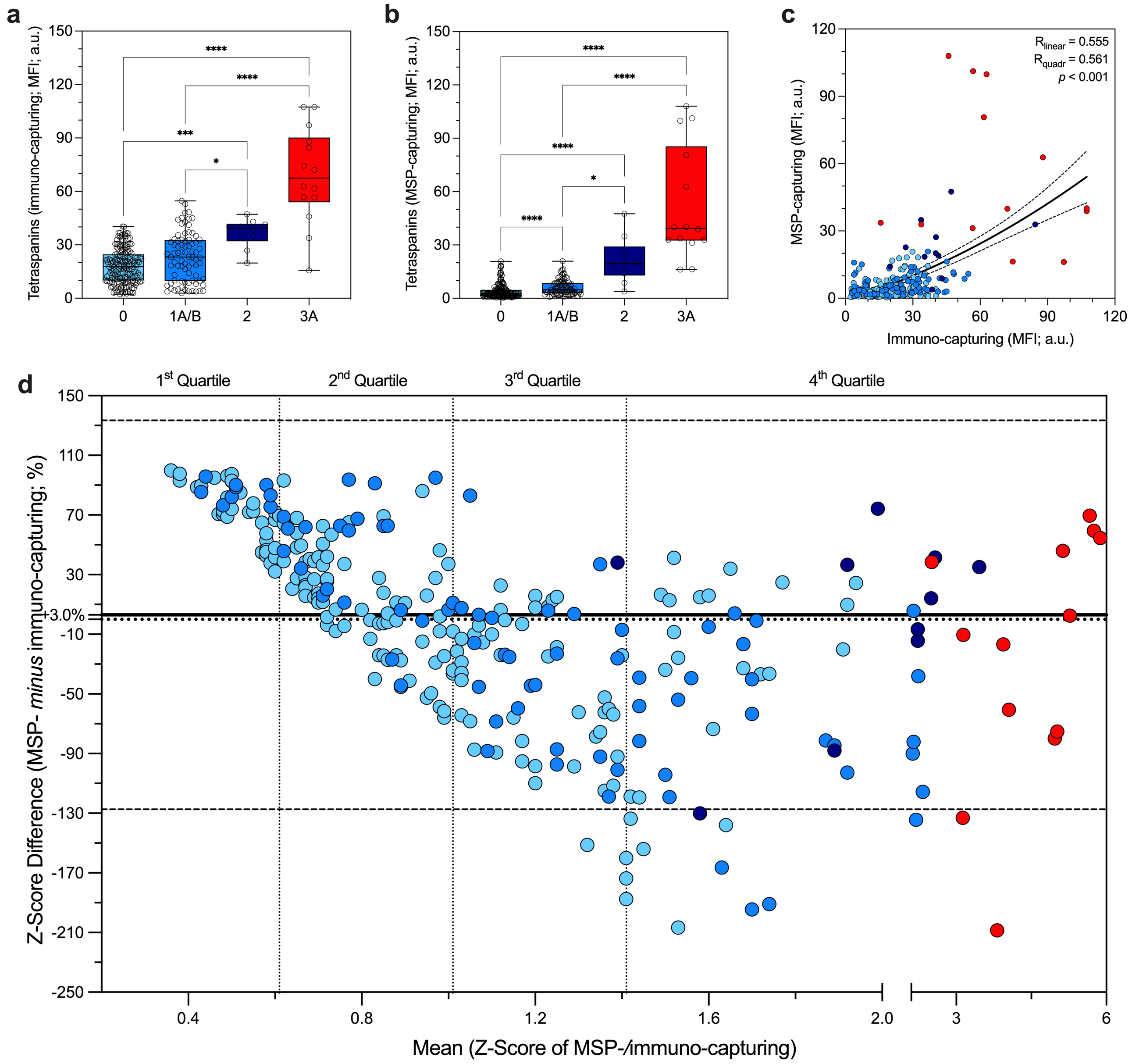

Bland-Altman analysis to compare levels of EV tetraspanins (CD9, CD63, and CD81) measured after immuno-capturing *versus* MSP-capturing. Grade of rejection is shown by color from 0 to 3A. Box plot and interquartile range are shown when appropriated. (**a**) Tetraspanins measured after immuno-capturing. (**b**) Tetraspanins measured after MSP-capturing. (**c**) Correlations between levels measured after immuno- and MSP-capturing; linear and quadratic correlation coefficients are reported together with *p*-value. (**d**) Bland-Altman plot of Z-score (see methods) comparing levels of EV-tetraspanins (a positive Z-score difference indicates an overestimate of MSP-capturing approach as compared to immuno-capturing). a.u., arbitrary unit; MFI, Median Fluorescence Intensity; MSP, Membrane Sensing Peptide. Source data and statistics are reported in Table S17.

Figure S6 – AI to detect rejection episodes: performance at tuning of the model

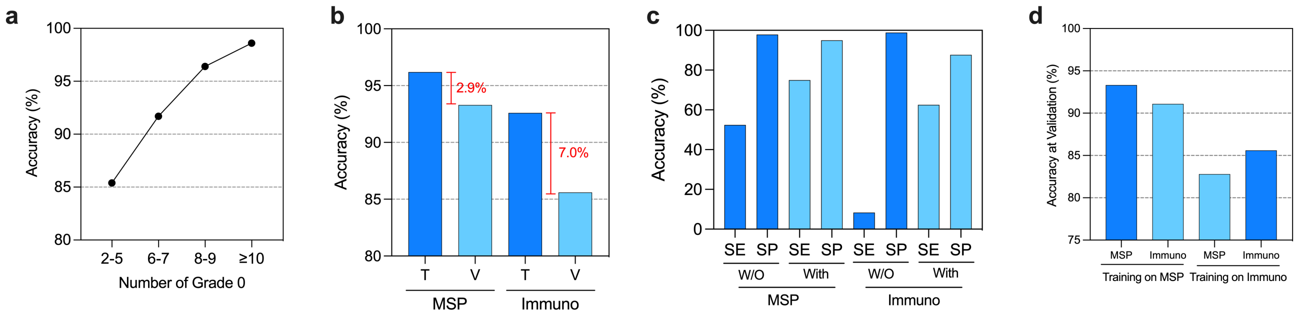

The tuning of the AI model includes: (**a**) Evaluation of accuracy at the increase of grade 0 episodes in graft trajectories; the x-axis reports the number of G0 of single patients. (**b**) Overfitting effect computed as difference between the accuracy at training (T) and at testing by leave-one-out validation algorithm (V) for EV profiling after capture by membrane-sensing peptide (MSP) *vs.* immuno-capture. (**c**) Evaluation of the effect of oversampling strategy on sensitivity (SE) and specificity (SP) of the AI model applied to EV profiling after MSP- *vs.* immuno-capturing; accuracy is reported with and without (W/O) the application of oversampling at the training of the model. (**d**) Transfer learning applied to our AI model; y-axis reports accuracy of the two methods for EV capture at validation, after training on data from EV profiling after MSP-capturing vs. immuno-capturing.

Table S1 – Protocols for EV isolation and surface antigens characterization

Step	MACSPlex immuno-capture (37 antigens)	MSP-capture custom panel (14 antigens)
**Rationale**	Immuno-capture exploiting antibody-covered multiplex beads; binding is driven by relatively low-affinity Ab–EV interactions that require prolonged incubation at 10 °C	Membrane-sensing peptide (MSP) covered beads exploiting high-avidity, electrostatic and hydrophobic interactions with EV lipid bilayer; binding occurs rapidly at room temperature
**Sample Preparation**	1 hour	1 hour
**EV Capture**	**10–12 h, 800 rpm, 10 °C** (overnight)	**2 h, 900 rpm, RT**
**Washing Step + Detection**	Two washes steps and incubation with detection mix (Ab anti-CD9/63/81) requiring 1.5 hours	Magnetic beads separation. Staining with Ab or MemGlow, single wash step; 1.5-2 hours.
**Cytometric analysis**	Acquisition on MACSQuant (approximately 1 minute per sample)	Acquisition on BD Symphony (approximately 1 minute per sample)
**Total turnaround**	**12–14 h**	**≈ 5 h**

Summary of main steps of the adopted protocols for EV isolation and surface antigens characterization.

Table S2 – EV surface profiling after MSP-capturing (14 antigens) according to rejection episodes

EV antigen	**Grade 0** (n=181)			**Grade 1A/B** (n=80)			**Grade 2** (n=10)			**Grade 3A** (n=14)			***P*-value**
	25th	*50th*	*75th*	*25th*	*50th*	*75th*	*25th*	*50th*	*75th*	*25th*	*50th*	*75th*	
**Tetrasp.**	1.3	2.7	4.8	3.0	4.8	8.7	12.8	19.5	29.1	32.5	39.4	85.5	**<0.001**
**CD2**	1.1	1.8	4.0	2.9	4.9	9.6	3.8	10.4	19.9	11.5	23.4	54.0	**<0.001**
**CD3**	0.5	0.8	1.7	1.3	2.3	5.4	1.8	6.0	14.5	11.8	17.7	30.9	**<0.001**
**CD4**	0.7	1.4	3.7	1.9	3.3	7.1	6.4	11.8	15.4	14.7	22.3	39.1	**<0.001**
**CD8**	1.3	2.5	5.5	2.9	5.7	11.0	7.0	20.1	37.7	10.1	35.2	67.0	**<0.001**
**CD19**	0.7	1.3	3.1	1.9	3.4	8.3	6.8	11.7	23.6	12.5	25.4	39.6	**<0.001**
**CD20**	1.1	2.0	4.6	3.1	4.6	9.6	9.2	19.4	27.5	9.4	31.0	64.0	**<0.001**
**CD24**	1.5	2.7	4.8	3.8	7.7	11.4	10.0	18.2	20.7	14.5	43.8	76.1	**<0.001**
**CD25**	0.9	1.5	3.9	2.2	3.9	7.3	7.7	13.3	22.7	17.7	28.7	46.6	**<0.001**
**CD45**	1.4	2.7	5.0	3.9	7.4	10.6	11.2	22.7	32.2	22.9	54.8	104.7	**<0.001**
**CD49e**	0.8	1.8	3.9	3.6	5.7	10.7	7.0	15.0	32.6	15.0	43.2	65.3	**<0.001**
**CD62p**	1.3	2.3	4.2	3.3	6.5	11.2	8.6	19.3	28.5	20.0	43.4	77.2	**<0.001**
**CD142**	2.2	4.1	8.9	7.8	11.3	15.2	27.0	40.9	65.6	54.0	83.6	109.0	**<0.001**
**CD209**	0.4	0.8	1.5	1.3	2.2	5.2	4.4	6.6	10.6	8.5	16.8	31.4	**<0.001**
**HLA-I**	3.4	6.0	11.9	11.3	14.5	23.3	15.0	64.3	97.0	75.5	120.8	152.3	**<0.001**

EV surface antigen profiling by flow cytometry after membrane sensing peptide (MSP)- capturing. Median fluorescence intensity (MFI; expressed as arbitrary unit, a.u.) is reported for tetraspanins (CD9, CD63, and CD81) and for a customized panel of 14 EV markers differentially expressed in rejecting recipients (see also Supplementary Data S2). Patients were stratified according to rejection grade (ACR from 0 to 3A). Data are expressed as median and interquartile range; a *P*-value less than 0.05 was considered significant and reported in bold.

Table S3 – EV surface profiling after MSP-capturing according to time-point of evaluation

EV antigen	After Surg. (n=17)			Grade 0 (n=109)			Pre 1A/B (n=48)			Pre 2-3A (n=7)			Grade 1A/B (n=80)			Grade 2-3A (n=24)			*P*-value
	25th	*50th*	*75th*	*25th*	*50th*	*75th*	*25th*	*50th*	*75th*	*25th*	*50th*	*75th*	*25th*	*50th*	*75th*	*25th*	*50th*	*75th*	
***Tetrasp.**	1.1	2.3	3.7	1.3	2.6	4.1	1.4	3.4	5.3	6.2	14.1	17.8	3.0	4.8	8.7	18.6	32.9	45.7	**<0.001**
***CD2**	1.0	1.5	2.9	0.9	1.7	3.2	1.2	2.2	5.2	2.8	3.8	9.0	2.9	4.9	9.6	8.2	17.1	33.3	**<0.001**
***CD3**	0.5	0.7	1.0	0.4	0.7	1.1	0.5	1.1	2.0	2.1	3.1	7.2	1.3	2.3	5.4	3.1	15.0	23.6	**<0.001**
***CD4**	0.6	1.0	1.9	0.6	1.3	3.5	1.0	2.3	4.3	1.9	6.9	8.3	1.9	3.3	7.1	7.9	18.5	28.5	**<0.001**
**CD8**	0.7	1.4	3.8	1.2	2.4	5.0	1.4	3.2	8.0	1.6	3.5	10.9	2.9	5.7	11.0	9.1	28.0	49.8	**<0.001**
***CD19**	0.6	0.9	1.8	0.6	1.0	2.1	1.0	2.4	4.5	2.2	3.1	10.9	1.9	3.4	8.3	7.9	16.2	32.1	**<0.001**
***CD20**	1.0	1.6	2.9	1.2	1.9	3.6	1.1	2.7	6.1	2.7	5.3	6.9	3.1	4.6	9.6	11.1	22.8	39.7	**<0.001**
***CD24**	1.5	2.1	3.5	1.4	2.9	4.7	1.3	2.5	5.3	3.4	5.8	7.6	3.8	7.7	11.4	14.3	20.5	50.6	**<0.001**
***CD25**	0.6	1.1	2.0	0.8	1.3	4.2	1.0	2.0	4.0	2.5	3.8	8.1	2.2	3.9	7.3	10.9	21.1	38.7	**<0.001**
***CD45**	0.9	1.8	4.2	1.3	2.3	4.4	1.8	4.0	6.9	2.9	6.1	13.5	3.9	7.4	10.6	13.8	34.9	81.3	**<0.001**
***CD49e**	0.8	1.4	3.9	0.8	1.7	3.4	0.7	2.1	6.0	2.7	7.4	10.4	3.6	5.7	10.7	11.9	25.6	50.2	**<0.001**
***CD62p**	0.9	1.5	2.8	1.3	2.2	3.4	1.5	3.2	5.5	3.7	4.4	10.7	3.3	6.5	11.2	15.9	28.6	58.5	**<0.001**
***CD142**	1.7	3.6	6.3	2.3	3.7	6.6	2.1	6.2	11.4	10.1	12.7	14.3	7.8	11.3	15.2	33.2	71.6	94.6	**<0.001**
***CD209**	0.5	0.6	1.0	0.4	0.7	1.2	0.4	1.0	1.9	1.8	5.2	6.2	1.3	2.2	5.2	5.3	10.7	18.3	**<0.001**
***HLA-I**	3.5	4.9	11.1	3.4	5.7	10.2	2.8	10.5	20.4	7.5	14.5	21.8	11.3	14.5	23.3	46.9	92.4	133.6	**<0.001**

EV surface antigen profiling by flow cytometry after membrane sensing peptide (MSP)- capturing. Median fluorescence intensity (MFI; expressed as arbitrary unit, a.u.) is reported for tetraspanins (CD9, CD63, and CD81) and for a customized panel of 14 EV markers differentially expressed in rejecting recipients (see also Supplementary Data S2). Patients were stratified according to the time-point of evaluation and rejection grade: after surgery (first sampling after heart transplant); grade 0 (non-rejecting patients); grade 1A/B; pre 1A/B (time-point of evaluation before a 1A/B diagnosis); grade 2-3A; pre 2-3A (time-point of evaluation before a 2-3A diagnosis). Data are expressed as median and interquartile range; a *P*-value less than 0.05 was considered significant and reported in bold. *EV markers significantly different between rejection diagnosis grade 0 and pre 2-3A.

Table S4 – EV surface profiling after MSP-capturing: sub-analysis on rejection episodes

EV antigen	**Grade 0-1A/B** (n=241)			**Pre 2-3A** (n=20)			**Grade 2-3A** (n=24)			**Overall *P*-value**	**Pairwise Comparison**		
	25th	*50th*	*75th*	*25th*	*50th*	*75th*	*25th*	*50th*	*75th*		0-1A/B *vs.* Pre 2-3A	0-1A/B *vs.* 2-3A	Pre 2-3A *vs.* 2-3A
**Tetrasp.**	1.6	3.2	6.0	2.8	5.1	9.2	18.6	32.9	45.7	**<0.001**	0.071	**<0.001**	**<0.001**
**CD2**	1.3	2.6	5.5	2.4	6.0	8.9	8.2	17.1	33.3	**<0.001**	**0.009**	**<0.001**	0.056
**CD3**	0.6	1.0	2.4	1.1	2.0	2.9	3.1	15.0	23.6	**<0.001**	0.283	**<0.001**	**<0.001**
**CD4**	0.9	2.0	5.0	2.0	3.2	7.1	7.9	18.5	28.5	**<0.001**	0.172	**<0.001**	**0.005**
**CD8**	1.5	3.1	7.9	2.4	6.3	13.8	9.1	28.0	49.8	**<0.001**	0.065	**<0.001**	0.068
**CD19**	0.8	1.8	4.0	1.2	2.0	4.6	7.9	16.2	32.1	**<0.001**	1.000	**<0.001**	**<0.001**
**CD20**	1.5	2.8	6.2	2.3	5.5	9.3	11.1	22.8	39.7	**<0.001**	0.077	**<0001**	**0.028**
**CD24**	1.9	3.7	6.4	3.1	8.1	12.1	14.3	20.5	50.6	**<0.001**	**0.010**	**<0.001**	**0.010**
**CD25**	1.0	2.2	4.9	1.4	3.2	8.2	10.9	21.1	38.7	**<0.001**	0.495	**<0.001**	**<0.001**
**CD45**	1.8	3.7	6.8	3.4	5.5	9.4	13.8	34.9	81.3	**<0.001**	0.170	**<0.001**	**0.001**
**CD49e**	1.1	2.8	5.6	3.7	7.5	13.0	11.9	25.6	50.2	**<0.001**	**0.003**	**<0.001**	**0.020**
**CD62p**	1.6	3.0	5.8	2.9	6.3	11.0	15.9	28.6	58.5	**<0.001**	**0.010**	**<0.001**	**0.007**
**CD142**	2.8	5.9	12.2	6.3	10.7	13.1	33.2	71.6	94.6	**<0.001**	0.125	**<0.001**	**0.002**
**CD209**	0.5	1.1	2.1	0.8	1.8	4.8	5.3	10.7	18.3	**<0.001**	0.127	**<0.001**	**0.004**
**HLA-I**	4.4	8.8	16.3	8.6	14.1	26.4	46.9	92.4	133.6	**<0.001**	0.079	**<0.001**	**0.001**

EV surface antigen profiling by flow cytometry after membrane sensing peptide (MSP)- capturing. Median fluorescence intensity (MFI; expressed as arbitrary unit, a.u.) is reported for tetraspanins (CD9, CD63, and CD81) and for a customized panel of 14 EV markers differentially expressed in rejecting recipients (see also Supplementary Data S2). Patients were stratified according to the time-point of evaluation and likelihood of rejection: grade 0-1A/B (non-rejecting patients and ACR 1A/B); pre 2-3A (time-point of evaluation before a 2-3A rejection diagnosis, when available); grade 2-3A. Data are expressed as median and interquartile range; a *P*-value less than 0.05 was considered significant and reported in bold.

Table S5 – Normalized levels of EV surface antigens after MSP-capturing during rejection episodes

EV antigen	**Grade 0** (n=181)			**Grade 1A/B** (n=80)			**Grade 2** (n=10)			**Grade 3A** (n=14)			***P*-value**
	25th	*50th*	*75th*	*25th*	*50th*	*75th*	*25th*	*50th*	*75th*	*25th*	*50th*	*75th*	
**Tetrasp.**	-21.0	17.7	151.7	48.9	220.3	396.1	525.5	973.1	2070.0	1470.3	2111.6	4890.6	**<0.001**
**CD2**	-36.0	20.2	126.2	67.3	246.6	685.7	52.3	633.3	1313.9	547.7	2161.8	4225.5	**<0.001**
**CD3**	-31.0	0.5	94.8	29.7	219.1	666.3	142.6	1094.0	2449.9	1503.2	4510.2	11131.9	**<0.001**
**CD4**	-13.3	30.9	244.5	62.5	215.4	968.0	328.2	842.6	1822.2	974.4	4577.0	23620.1	**<0.001**
**CD8**	-24.6	33.8	172.3	22.7	314.2	1031.7	381.5	830.5	2254.7	394.0	1867.8	3365.7	**<0.001**
**CD19**	-12.9	29.9	183.9	64.1	298.5	762.3	489.7	954.3	2879.9	1127.4	2101.4	6987.8	**<0.001**
**CD20**	-37.1	10.2	107.5	50.3	242.0	729.6	246.1	856.4	1727.0	260.0	1788.0	4136.7	**<0.001**
**CD24**	-30.4	0.0	62.7	77.0	243.7	499.2	205.4	672.3	1302.2	745.1	2145.9	6475.5	**<0.001**
**CD25**	-22.0	11.7	136.9	39.6	201.0	489.5	335.0	981.9	2756.0	1254.9	1827.9	5301.4	**<0.001**
**CD45**	-31.3	10.8	102.9	57.2	260.1	502.9	401.4	871.8	1802.8	976.1	2971.4	5035.7	**<0.001**
**CD49e**	-33.9	4.3	96.1	135.1	276.5	891.6	412.6	921.8	1424.0	898.7	1900.3	3461.7	**<0.001**
**CD62p**	-26.2	1.9	74.3	85.9	244.0	543.0	361.6	733.5	2224.7	1086.5	2286.9	3300.8	**<0.001**
**CD142**	-20.2	3.0	60.9	43.5	255.4	535.3	250.9	1071.4	1884.4	1093.6	3472.8	4290.2	**<0.001**
**CD209**	-32.1	2.5	91.5	47.4	271.5	1451.9	225.9	1018.8	1664.2	1043.1	3822.0	7112.4	**<0.001**
**HLA-I**	-36.5	0.0	72.4	57.5	205.9	382.5	237.8	575.7	1760.1	357.4	2603.5	4502.6	**<0.001**

EV surface antigen profiling by flow cytometry after membrane sensing peptide (MSP)- capturing. Median fluorescence intensity (MFI; expressed as arbitrary unit, a.u.) is reported for tetraspanins and 14 differentially expressed EV markers, after normalization for median levels of each antigen in correspondence of grade 0 episodes for each single patient, and reported as percentage of variation (i.e., for patient #1, we calculated median MFI of CD2 for grade 0 and then normalized each CD2 measurement for median $CD2_{G0\text{-}ID\#1}$ according to following equation: $Normalized\ Delta\text{-}CD2_{ID\#1}\ [\%] = [(CD2_{ID\#1}\ [a.u.])-(MedianCD2_{G0\text{-}ID\#1}\ [a.u.])]/\ (MedianCD2_{G0\text{-}ID\#1}\ [a.u.])*100$). Patients were stratified according to rejection grade (from 0 to 3A). Data are expressed as median and interquartile range; a *P*-value less than 0.05 was considered significant and reported in bold.

Table S6 – Normalized levels of EV surface antigens after MSP-capturing at time-point analysis

EV antigen	**After Surg.** (n=17)			**Grade 0** (n=109)			**Pre 1A/B** (n=48)			**Pre 2-3A** (n=7)			**Grade 1A/B** (n=80)			**Grade 2-3A** (n=24)			*P*-value
	25th	*50th*	*75th*	*25th*	*50th*	*75th*	*25th*	*50th*	*75th*	*25th*	*50th*	*75th*	*25th*	*50th*	*75th*	*25th*	*50th*	*75th*	
**Tetrasp.**	-47.4	9.7	103.9	-14.2	6.0	91.8	-17.7	85.2	198.0	410.7	538.9	812.4	48.9	220.3	396.1	777.0	1702.7	2423.0	**<0.001**
**CD2**	-48.0	-0.2	85.8	-38.4	1.5	72.5	-26.9	75.4	243.0	16.0	285.4	646.4	67.3	246.6	685.7	396.4	1262.8	3027.1	**<0.001**
**CD3**	-53.0	-10.8	80.0	-30.9	0.0	39.1	-28.9	65.1	197.1	69.1	352.8	1549.1	29.7	219.1	666.3	544.7	2257.5	5745.1	**<0.001**
**CD4**	-28.2	-16.4	157.1	-11.5	14.6	111.0	2.2	131.0	499.5	4.7	422.8	1154.3	62.5	215.4	968.0	681.8	1644.6	5632.8	**<0.001**
**CD8**	-55.9	-18.9	101.6	-20.1	19.6	99.2	-24.3	108.4	600.7	-8.2	81.7	579.8	22.7	314.2	1031.7	458.2	1365.7	3074.3	**<0.001**
**CD19**	-38.5	21.5	117.4	-19.9	9.7	62.2	13.0	127.5	455.3	112.9	519.2	1011.0	64.1	298.5	762.3	684.2	1626.7	6133.9	**<0.001**
**CD20**	-59.5	-1.6	57.2	-36.2	0.0	60.0	-39.1	56.8	488.2	-0.9	134.9	554.5	50.3	242.0	729.6	271.4	1214.7	3533.4	**<0.001**
**CD24**	-46.2	-29.3	33.5	-26.8	0.0	40.6	-41.8	20.5	116.7	78.0	195.9	355.0	77.0	243.7	499.2	554.3	1433.7	3007.5	**<0.001**
**CD25**	-42.7	-22.1	21.7	-19.8	11.1	82.8	-21.1	21.7	227.1	37.5	179.9	587.5	39.6	201.0	489.5	587.2	1522.2	3754.7	**<0.001**
**CD45**	-61.1	9.6	148.2	-31.3	0.0	37.3	-36.3	77.9	246.0	108.8	218.5	291.2	57.2	260.1	502.9	528.1	1776.5	4034.3	**<0.001**
**CD49e**	-64.0	6.3	114.1	-31.2	0.0	52.8	-46.3	62.2	223.3	5.5	441.1	1122.8	135.1	276.5	891.6	664.7	1223.9	2575.1	**<0.001**
**CD62p**	-60.2	-20.9	57.4	-21.6	0.0	44.2	-30.0	37.9	151.7	-7.3	317.7	525.7	85.9	244.0	543.0	683.7	1349.9	2568.7	**<0.001**
**CD142**	-60.7	-16.8	28.1	-19.4	0.0	40.0	-17.0	44.4	228.5	28.9	190.5	552.5	43.5	255.4	535.3	476.1	1540.5	3638.1	**<0.001**
**CD209**	-60.6	-25.1	126.0	-32.1	0.0	38.0	-27.0	72.1	260.4	176.8	480.4	994.6	47.4	271.5	1451.9	699.9	1701.0	5720.9	**<0.001**
**HLA-I**	-61.2	-19.5	78.8	-36.5	0.0	40.4	-30.7	4.3	223.3	39.6	177.6	373.5	57.5	205.9	382.5	261.3	1278.9	4069.3	**<0.001**

EV surface antigen profiling by flow cytometry after membrane sensing peptide (MSP)- capturing. Median fluorescence intensity (MFI; expressed as arbitrary unit, a.u.) is reported for tetraspanins and 14 differentially expressed EV markers, after normalization for median levels of each antigen in correspondence of grade 0 episodes for each single patient, and reported as percentage of variation (i.e., for patient #1, we calculated median MFI of CD2 for grade 0 and then normalized each CD2 measurement for median $CD2_{G0\text{-}ID\#1}$ according to following equation: $Normalized\ Delta\text{-}CD2_{ID\#1}\ [\%] = [(CD2_{ID\#1}\ [a.u.])-(MedianCD2_{G0\text{-}ID\#1}\ [a.u.])]/\ (MedianCD2_{G0\text{-}ID\#1}\ [a.u.])*100$). Patients were stratified according to the time-point of evaluation and rejection grade: after surgery (first sampling after heart transplant); grade 0 (non-rejecting patients); rejection grade 1A/B; pre 1A/B (time-point of evaluation before a 1A/B ACR diagnosis); rejection grade 2-3A; pre 2-3A (time-point of evaluation before a 2-3A ACR diagnosis).Data are expressed as median and interquartile range; a *P*-value less than 0.05 was considered significant and reported in bold.

Table S7 – Association of single EV surface antigens with the diagnosis of rejection

EV antigen	Absolute Values (a.u.)			Delta Variation (%)		
	OR	*P-value*	*95% CI*	*OR*	*P-value*	*95% CI*
**Tetrasp.**	1.402	**<0.001**	1.228 – 1.600	1.340	**<0.001**	1.223 – 1.468
**CD2**	1.229	**<0.001**	1.140 – 1.324	1.112	**<0.001**	1.066 – 1.159
**CD3**	1.112	**<0.001**	1.060 – 1.165	1.035	**<0.001**	1.019 – 1.050
**CD4**	1.140	**<0.001**	1.085 – 1.197	1.007	**<0.001**	1.002 – 1.013
**CD8**	1.077	**<0.001**	1.050 – 1.104	1.035	**<0.001**	1.016 – 1.055
**CD19**	1.134	**<0.001**	1.085 – 1.186	1.049	**<0.001**	1.029 – 1.070
**CD20**	1.196	**<0.001**	1.125 – 1.271	1.139	**<0.001**	1.084 – 1.197
**CD24**	1.275	**<0.001**	1.170 – 1.390	1.071	**<0.001**	1.037 – 1.105
**CD25**	1.259	**<0.001**	1.165 – 1.361	1.207	**<0.001**	1.124 – 1.296
**CD45**	1.240	**<0.001**	1.144 – 1.344	1.267	**<0.001**	1.167 – 1.376
**CD49e**	1.258	**<0.001**	1.150 – 1.377	1.159	**<0.001**	1.099 – 1.223
**CD62p**	1.270	**<0.001**	1.167 – 1.382	1.212	**<0.001**	1.141 – 1.289
**CD142**	1.161	**<0.001**	1.092 – 1.235	1.369	**<0.001**	1.222 – 1.534
**CD209**	1.252	**<0.001**	1.158 – 1.354	1.058	**<0.001**	1.035 – 1.081
**HLA-I**	1.097	**<0.001**	1.060 – 1.136	1.269	**<0.001**	1.156 – 1.393

Univariate regression analysis of EV profiling after MSP-capturing (absolute values and delta variation) and rejection status. Odds ratio (OR) with 95% confidence intervals and *p*-value are reported for each comparison. An OR higher than 1 indicates an increased likelihood of ACR grade 2-3A (n=24), an OR less than 1 a decreased likelihood. Referral group is composed by non-rejecting patients and grade 1A/B ACR (n=261). *P*-values of less than 0.05 were considered significant.

Table S8 – Diagnostic performance of single EV surface antigens

EV antigen	Absolute Values (a.u.)						Delta Variation (%)					
	Area under the Curve			Sensitivity/Specificity			Area under the Curve			Sensitivity/Specificity		
	AUC	*P-value**	*95% CI*	*Cut-off (≥)*	*Sens (%)*	*Spec (%)*	*AUC*	*P-value**	*95% CI*	*Cut-off (≥)*	*Sens (%)*	*Spec (%)*
**Tetrasp.**	0.972	**<0.001**	0.937 – 1.000	14.1	91.7	95.8	0.937	**<0.001**	0.869 – 1.000	482.7	91.7	88.9
**CD2**	0.875	**<0.001**	0.789 – 0.962	7.3	83.3	84.3	0.847	**<0.001**	0.758 – 0.937	386.5	79.2	81.2
**CD3**	0.865	**<0.001**	0.780 – 0.950	4.1	75.0	86.2	0.859	**<0.001**	0.773 – 0.945	423.9	80.2	81.6
**CD4**	0.880	**<0.001**	0.803 – 0.957	11.0	70.8	93.5	0.852	**<0.001**	0.786 – 0.918	296.7	87.5	71.3
**CD8**	0.838	**<0.001**	0.740 – 0.936	12.8	70.8	87.7	0.785	**<0.001**	0.680 – 0.890	448.7	79.2	74.3
**CD19**	0.909	**<0.001**	0.858 – 0.960	4.4	91.7	77.0	0.893	**<0.001**	0.847 – 0.939	249.4	100.0	71.9
**CD20**	0.862	**<0.001**	0.767 – 0.958	10.8	79.2	89.3	0.839	**<0.001**	0.759 – 0.920	154.8	91.7	66.3
**CD24**	0.930	**<0.001**	0.872 – 0.987	10.7	87.5	89.7	0.885	**<0.001**	0.809 – 0.961	253.9	91.7	80.1
**CD25**	0.924	**<0.001**	0.863 – 0.986	9.0	83.3	89.7	0.906	**<0.001**	0.842 – 0.971	426.6	87.5	83.1
**CD45**	0.928	**<0.001**	0.861 – 0.994	12.7	87.5	92.0	0.929	**<0.001**	0.883 – 0.975	429.2	83.3	86.6
**CD49e**	0.930	**<0.001**	0.879 – 0.981	11.1	79.2	91.2	0.898	**<0.001**	0.844 – 0.951	453.4	87.5	82.8
**CD62p**	0.933	**<0.001**	0.876 – 0.990	15.8	79.2	96.9	0.903	**<0.001**	0.832 – 0.974	395.0	91.7	83.1
**CD142**	0.917	**<0.001**	0.840 – 0.994	31.0	79.2	99.2	0.912	**<0.001**	0.845 – 0.978	917.3	70.8	98.5
**CD209**	0.898	**<0.001**	0.828 – 0.968	3.3	87.5	85.4	0.891	**<0.001**	0.839 – 0.944	688.1	79.2	86.2
**HLA-I**	0.933	**<0.001**	0.873 – 0.993	40.0	83.3	96.9	0.884	**<0.001**	0.804 – 0.965	186.9	95.8	73.9

ROC curves analysis of EV profiling after MSP-capturing (absolute values and delta variation) and rejection status: grade 2-3A ACR (n=24) *vs.* non-rejecting patients and grade 1A/B ACR (n=261). The table reports area under the curve (AUC), 95% confidence intervals (CI), asymptotical significance (*p*-value for the comparison with the referral line, AUC=0.5), cut-off corresponding to the highest accuracy according to the Youden index, sensitivity, and specificity. *P*-values of less than 0.05 were considered significant and highlighted in bold characters.

Table S9 – Correlation analysis of EV profiling and biochemical patients' profile

Correlation analysis	Tetra	CD2	CD3	CD4	CD8	CD19	CD20	CD24	CD25	CD45	CD49e	CD62P	CD142	CD209	HLA-I
WBC (*10.9/L)	-0.009	-0.026	-0.052	-0.025	-0.102	-0.019	-0.018	-0.016	-0.050	-0.003	-0.014	-0.016	0.001	-0.044	0.023
	0.885	0.667	0.384	0.679	0.088	0.747	0.759	0.791	0.401	0.965	0.811	0.786	0.986	0.466	0.704
Neutrophils (*10.9/L)	-0.020	-0.027	-0.055	-0.035	-0.117	-0.041	-0.033	-0.024	-0.064	-0.004	-0.019	-0.025	-0.009	-0.050	0.017
	0.744	0.649	0.365	0.565	0.051	0.492	0.587	0.695	0.285	0.947	0.748	0.679	0.877	0.403	0.777
Lymphocytes (*10.9/L)	-0.034	-0.076	-0.067	-0.030	0.014	0.024	0.004	-0.062	0.001	-0.078	-0.075	-0.035	-0.042	-0.031	-0.062
	0.568	0.208	0.263	0.614	0.810	0.688	0.949	0.304	0.985	0.194	0.213	0.565	0.488	0.604	0.300
Monocytes (*10.9/L)	0.078	0.062	-0.020	0.057	0.011	0.079	0.046	0.049	-0.009	0.072	0.055	0.045	0.076	0.030	0.087
	0.192	0.304	0.738	0.341	0.861	0.191	0.447	0.420	0.883	0.231	0.360	0.453	0.205	0.624	0.150
Eosinophils (*10.9/L)	-0.018	-0.017	-0.053	0.024	-0.011	-0.003	-0.016	-0.015	-0.017	0.027	-0.018	-0.024	0.027	-0.027	0.045
	0.767	0.774	0.379	0.685	0.850	0.960	0.788	0.807	0.776	0.654	0.764	0.688	0.660	0.657	0.457
Basophils (*10.9/L)	0.218	0.282	0.175	0.264	0.236	0.299	0.280	0.313	0.136	0.230	0.286	0.225	0.170	0.277	0.156
	**<0.001**	**<0.001**	**0.003**	**<0.001**	**<0.001**	**<0.001**	**<0.001**	**<0.001**	**0.023**	**<0.001**	**<0.001**	**<0.001**	**0.005**	**<0.001**	**0.009**
ASL (U/L)	0.089	0.049	-0.012	0.084	-0.008	0.047	0.102	0.023	0.010	0.081	0.032	0.049	0.072	0.044	0.107
	0.135	0.411	0.836	0.159	0.897	0.433	0.085	0.703	0.873	0.171	0.591	0.413	0.226	0.457	0.071
ALT (U/L)	0.053	0.044	0.009	0.090	0.005	0.038	0.096	0.021	0.005	0.060	0.033	0.051	0.045	0.057	0.057
	0.376	0.460	0.877	0.132	0.936	0.525	0.105	0.721	0.930	0.313	0.583	0.389	0.450	0.335	0.335
GGT (U/L)	0.051	0.018	-0.011	0.007	-0.041	0.013	0.036	0.036	0.011	0.057	0.036	0.020	0.047	0.017	0.062
	0.409	0.770	0.857	0.913	0.504	0.829	0.562	0.559	0.858	0.348	0.559	0.740	0.443	0.785	0.309
CPK (U/L)	-0.105	-0.095	-0.115	-0.071	-0.143	-0.041	-0.064	-0.092	-0.048	-0.083	-0.093	-0.102	-0.091	-0.091	-0.057
	0.126	0.170	0.094	0.304	**0.037**	0.556	0.351	0.183	0.489	0.231	0.179	0.138	0.187	0.186	0.408
Creatinine (mg/dL)	-0.063	-0.073	-0.097	-0.025	-0.021	-0.044	-0.111	-0.048	-0.071	-0.065	-0.085	-0.064	-0.046	-0.073	-0.069
	0.291	0.218	0.104	0.680	0.728	0.459	0.061	0.420	0.232	0.273	0.154	0.283	0.443	0.221	0.245
eGFR (mL/min)	0.110	0.125	0.167	0.072	0.033	0.068	0.147	0.093	0.116	0.124	0.142	0.120	0.081	0.126	0.134
	0.077	**0.043**	**0.007**	0.248	0.598	0.277	**0.018**	0.135	0.062	**0.046**	**0.022**	0.053	0.193	**0.041**	**0.031**

Correlation analysis between biochemical parameters and EV profiling after MSP-capturing (n=285); Pearson's R coefficient (above) and *p*-value (below) are reported for each comparison. WBC, White Blood Cells; eGFR, estimated Glomerular Filtration Rate; *P*-values of less than 0.05 were considered significant and highlighted in red and bold characters.

Table S10 – Association analysis of EV profiling with endomyocardial biopsy characteristics

Regression analysis	Tetra	CD2	CD3	CD4	CD8	CD19	CD20	CD24	CD25	CD45	CD49e	CD62P	CD142	CD209	HLA-I
Fibrosis	0.98<br>(0.96-1.01)<br>0.156	0.98<br>(0.96-1.01)<br>0.275	0.96<br>(0.92-1.00)<br>0.071	0.97<br>(0.93-1.01)<br>0.093	0.99<br>(0.98-1.01)<br>0.411	0.98<br>(0.95-1.01)<br>0.157	0.97<br>(0.944-1.01)<br>0.096	0.98<br>(0.96-1.01)<br>0.171	0.98<br>(0.95-1.01)<br>0.188	0.99<br>(0.97-1.01)<br>0.156	0.98<br>(0.96-1.01)<br>0.162	0.99<br>(0.97-1.01)<br>0.249	0..99<br>(0.98-1.01)<br>0.332	0.98<br>(0.94-1.02)<br>0.256	0.99<br>(0.98-1.00)<br>0.107
Inflammatory infiltrate	1.10<br>(1.05-1.15)<br>**<0.001**	1.16<br>(1.09-1.23)<br>**<0.001**	1.15<br>(1.09-1.23)<br>**<0.001**	1.07<br>(1.03-1.11)<br>**0.001**	1.06<br>(1.03-1.08)<br>**<0.001**	1.08<br>(1.04-1.12)<br>**<0.001**	1.07<br>(1.03-1.12)<br>**<0.001**	1.14<br>(1.08-1.20)<br>**<0.001**	1.12<br>(1.06-1.18)<br>**<0.001**	1.07<br>(1.03-1.11)<br>**0.001**	1.13<br>(1.07-1.20)<br>**<0.001**	1.13<br>(1.07-1.19)<br>**<0.001**	1.06<br>(1.03-1.10)<br>**<0.001**	1.22<br>(1.12-1.32)<br>**<0.001**	1.04<br>(1.02-1.06)<br>**<0.001**
Myocytolisis	1.07<br>(1.04-1.11)<br>**<0.001**	1.07<br>(1.03-1.11)<br>**0.001**	1.07<br>(1.03-1.11)<br>**<0.001**	1.07<br>(1.03-1.11)<br>**<0.001**	1.05<br>(1.02-1.07)<br>**<0.001**	1.07<br>(1.04-1.11)<br>**<0.001**	1.06<br>(1.03-1.09)<br>**<0.001**	1.05<br>(1.02-1.07)<br>**0.001**	1.06<br>(1.03-1.09)<br>**<0.001**	1.04<br>(1.02-1.07)<br>**<0.001**	1.06<br>(1.03-1.10)<br>**<0.001**	1.06<br>(1.03-1.09)<br>**<0.001**	1.03<br>(1.02-1.05)<br>**<0.001**	1.14<br>(1.07-1.21)<br>**<0.001**	1.02<br>(1.01-1.03)<br>**<0.001**
Myocytes necrosis	1.11<br>(1.07-1.16)<br>**<0.001**	1.09<br>(1.04-1.14)<br>**<0.001**	1.07<br>(1.03-1.11)<br>**<0.001**	1.08<br>(1.04-1.13)<br>**<0.001**	1.06<br>(1.03-1.08)<br>**<0.001**	1.08<br>(1.04-1.12)<br>**<0.001**	1.08<br>(1.04-1.12)<br>**<0.001**	1.06<br>(1.02-1.09)<br>**<0.001**	1.11<br>(1.06-1.16)<br>**<0.001**	1.06<br>(1.03-1.08)<br>**<0.001**	1.08<br>(1.04-1.11)<br>**<0.001**	1.08<br>(1.04-1.11)<br>**<0.001**	1.05<br>(1.03-1.06)<br>**<0.001**	1.15<br>(1.08-1.22)<br>**<0.001**	1.03<br>(1.02-1.05)<br>**<0.001**
Ischemic damage	1.01<br>(0.99-1.03)<br>0.342	1.01<br>(0.99-1.03)<br>0.235	1.02<br>(1.01-1.05)<br>**0.041**	1.01<br>(0.99-1.03)<br>0.290	1.00<br>(0.98-1.02)<br>0.971	1.01<br>(0.99-1.024)<br>0.427	1.00<br>(0.98-1.03)<br>0.949	1.00<br>(0.99-1.02)<br>0.636	1.01<br>(0.99-1.04)<br>0.393	1.01<br>(0.99-1.02)<br>0.228	1.01<br>(0.99-1.02)<br>0.258	1.01<br>(0.99-1.03)<br>0.236	1.01<br>(0.99-1.02)<br>0.453	1.02<br>(0.99-1.04)<br>0.192	1.00<br>(0.99-1.01)<br>0.508
Edema	1.02<br>(1.01-1.04)<br>**0.022**	1.03<br>(1.01-1.06)<br>**0.018**	1.02<br>(0.99-1.04)<br>0.174	1.02<br>(1.00-1.04)<br>0.058	1.02<br>(0.99-1.04)<br>0.060	1.02<br>(1.00-1.04)<br>0.077	1.03<br>(1.01-1.05)<br>**0.016**	1.02<br>(1.00-1.03)<br>**0.048**	1.02<br>(0.99-1.04)<br>0.220	1.02<br>(1.01-1.03)<br>**0.029**	1.02<br>(1.00-1.03)<br>0.060	1.02<br>(1.00-1.03)<br>0.059	1.01<br>(1.01-1.03)<br>**0.029**	1.03<br>(1.00-1.05)<br>0.061	1.01<br>(1.01-1.02)<br>**0.009**
Vasculitis	1.02<br>(1.00-1.04)<br>0.128	1.01<br>(0.99-1.03)<br>0.389	1.02<br>(1.01-1.05)<br>**0.028**	1.00<br>(0.98-1.02)<br>0.830	1.01<br>(0.99-1.03)<br>0.219	1.00<br>(0.98-1.02)<br>0.721	1.01<br>(0.99-1.03)<br>0.233	1.01<br>(0.99-1.02)<br>0.310	1.03<br>(1.01-1.06)<br>**0.016**	1.01<br>(0.99-1.02)<br>0.296	1.01<br>(0.99-1.02)<br>0.502	1.01<br>(0.99-1.03)<br>0.496	1.01<br>(1.01-1.03)<br>**0.036**	1.01<br>(0.98-1.03)<br>0.565	1.01<br>(1.01-1.02)<br>**0.008**
Micro-vasculopathy	1.00<br>(0.98-1.03)<br>0.990	1.00<br>(0.97-1.03)<br>0.877	1.01<br>(0.99-1.03)<br>0.435	1.01<br>(0.99-1.03)<br>0.631	1.00<br>(0.97-1.02)<br>0.742	1.00<br>(0.98-1.02)<br>0.844	1.00<br>(0.97-1.03)<br>0.802	1.00<br>(0.98-1.02)<br>0.962	1.02<br>(0.99-1.05)<br>0.129	1.00<br>(0.97-1.02)<br>0.678	1.00<br>(0.98-1.02)<br>0.829	1.00<br>(0.98-1.02)<br>0.726	1.00<br>(0.98-1.01)<br>0.602	1.01<br>(0.98-1.03)<br>0.619	0.99<br>(0.98-1.01)<br>0.301
Vessel thrombosis	0.87<br>(0.59-1.29)<br>0.494	1.02<br>(0.97-1.09)<br>0.466	1.02<br>(0.97-1.07)<br>0.446	1.00<br>(0.92-1.08)<br>0.992	0.99<br>(0.90-1.09)<br>0.856	1.01<br>(0.97-1.05)<br>0.589	0.98<br>(0.83-1.15)<br>0.792	1.01<br>(0.97-1.05)<br>0.749	1.03<br>(0.98-1.09)<br>0.186	1.00<br>(0.94-1.06)<br>0.952	1.00<br>(0.93-1.08)<br>0.967	1.00<br>(0.91-1.09)<br>0.938	1.00<br>(0.94-1.06)<br>0.903	1.01<br>(0.96-1.07)<br>0.641	0.99<br>(0.93-1.05)<br>0.745
Quilty effect	0.99<br>(0.95-1.03)<br>0.642	0.97<br>(0.89-1.05)<br>0.403	0.99<br>(0.95-1.04)<br>0.778	0.97<br>(0.90-1.05)<br>0.454	1.01<br>(0.98-1.03)<br>0.602	0.97<br>(0.91-1.04)<br>0.410	0.99<br>(0.94-1.04)<br>0.620	1.00<br>(0.97-1.03)<br>0.829	0.96<br>(0.88-1.04)<br>0.331	0.99<br>(1.095-1.03)<br>0.530	0.99<br>(0.94-1.04)<br>0.580	0.99<br>(0.94-1.03)<br>0.549	0.99<br>(0.96-1.02)<br>0.499	0.93<br>(0.80-1.07)<br>0.306	0.99<br>(0.96-1.01)<br>0.544
Peri-operative damage	1.01<br>(0.99-1.03)<br>0.554	1.01<br>(0.99-1.03)<br>0.433	1.02<br>(1.01-1.05)<br>**0.037**	1.01<br>(0.99-1.03)<br>0.430	1.00<br>(0.98-1.02)<br>0.928	1.01<br>(0.99-1.02)<br>0.486	1.01<br>(0.98-1.03)<br>0.648	1.00<br>(0.99-1.02)<br>0.673	1.01<br>(0.98-1.04)<br>0.516	1.01<br>(0.99-1.02)<br>0.498	1.01<br>(0.99-1.02)<br>0.317	1.01<br>(0.99-1.03)<br>0.355	1.00<br>(0.99-1.02)<br>0.808	1.01<br>(0.99-1.04)<br>0.239	1.00<br>(0.99-1.01)<br>0.724

Univariate regression analysis assesses associations between characteristics of endomyocardial biopsy and EV profiling after MSP-capturing (n=285). Odds ratio (OR) with 95% confidence intervals (above) and *p*-value (below) are reported for each comparison. An OR higher than 1 indicates an increased likelihood of each considered single parameter, an OR less than 1 a decreased likelihood. *P*-values of less than 0.05 were considered significant and highlighted in red and bold characters.

Table S11 – Association of EV markers and rejection episodes independently from immunosuppressive treatment

EV antigen (MFI; a.u.)	Multivariate Analysis [correction for immunosuppressive regimen]		
	OR	*P-value*	*95% CI*
**Tetrasp.**	1.413	**<0.001**	1.234-1.618
**CD2**	1.231	**<0.001**	1.141-1.329
**CD3**	1.114	**<0.001**	1.062-1.168
**CD4**	1.140	**<0.001**	1.086-1.198
**CD8**	1.077	**<0.001**	1.049-1.104
**CD19**	1.134	**<0.001**	1.085-1.186
**CD20**	1.195	**<0.001**	1.125-1.270
**CD24**	1.285	**<0.001**	1.178-1.403
**CD25**	1.261	**<0.001**	1.166-1.364
**CD45**	1.241	**<0.001**	1.147-1.344
**CD49e**	1.275	**<0.001**	1.160-1.401
**CD62p**	1.281	**<0.001**	1.173-1.399
**CD142**	1.162	**<0.001**	1.094-1.235
**CD209**	1.256	**<0.001**	1.161-1.359
**HLA-I**	1.097	**<0.001**	1.060-1.135

Multivariate regression analysis of EV profiling after MSP-capturing (median fluorescence intensity, MFI; expressed as arbitrary unit, a.u.), after correction for immunosuppressive treatment (cyclosporine-based *vs.* tacrolimus-based regimen). Odds ratio (OR) with 95% confidence intervals and *p*-value are reported for each comparison. An OR higher than 1 indicates an increased likelihood of grade 2-3A ACR (n=24), an OR less than 1 a decreased likelihood. Referral group is composed by non-rejecting patients and grade 1A/B ACR (n=261). *P*-values of less than 0.05 were considered significant.

Table S12 – EV surface profiling after MSP-capturing: sub-analysis on immunosuppressive treatment (before vs. after a change in immunosuppressive regimen)

**EV antigen**	**Before** (n= 27)			**After** (n=27)			***P*-value**
	25th	*50th*	*75th*	*25th*	*50th*	*75th*	
**Tetrasp.**	8.2	25.0	39.7	1.7	3.8	8.1	**<0.001**
**CD2**	5.0	12.6	22.8	1.6	3.3	8.5	**<0.001**
**CD3**	2.0	11.8	17.0	0.5	1.1	2.1	**<0.001**
**CD4**	4.2	13.0	20.2	1.1	2.4	3.7	**<0.001**
**CD8**	2.5	13.9	41.5	1.7	4.9	14.8	0.053
**CD19**	3.2	14.4	30.7	1.0	1.3	4.1	**<0.001**
**CD20**	5.2	16.7	29.1	1.9	3.0	6.8	**<0.001**
**CD24**	11.6	16.0	33.7	2.4	3.9	8.7	**<0.001**
**CD25**	5.2	17.7	30.0	1.3	1.5	4.2	**<0.001**
**CD45**	7.5	14.6	38.1	1.0	3.4	8.5	**<0.001**
**CD49e**	12.9	25.5	56.3	2.5	4.0	7.6	**<0.001**
**CD62p**	8.9	17.6	38.6	1.8	2.9	8.6	**<0.001**
**CD142**	11.7	48.7	84.8	2.9	7.3	11.4	**<0.001**
**CD209**	2.7	10.0	15.3	0.6	1.2	2.4	**<0.001**
**HLA-I**	20.6	77.8	121.2	4.9	10.0	15.9	**<0.001**

EV surface antigen profiling by flow cytometry after membrane sensing peptide (MSP)- capturing, before and after a change in immunosuppressive regimen (Wilcoxon matched-pairs signed rank test). Median fluorescence intensity (MFI; expressed as arbitrary unit, a.u.) is reported for tetraspanins (CD9, CD63, and CD81) and for a customized panel of 14 EV markers differentially expressed in rejecting recipients (see also Supplementary Data S2). Data are expressed as median and interquartile range; a *P*-value less than 0.05 was considered significant and reported in bold.

Table S13 – Likelihood of rejection according to AI and EV profiling after MSP-capturing

Model Coefficient	Total	Grade 0		Grade 1A/B		Grade 2		Grade 3A	
		n	%	n	%	n	%	n	%
0.00-0.10	181	135	74.6	46	25.4	0	0.0	0	0.0
0.11-0.20	25	18	72.0	7	28.0	0	0.0	0	0.0
0.21-0.30	25	16	64.0	8	32.0	0	0.0	1	4.0
0.31-0.40	10	4	40.0	4	40.0	1	10.0	1	10.0
0.41-0.50	13	4	30.8	6	46.2	2	15.4	1	7.7
0.51-0.60	6	2	33.3	3	50.0	1	16.7	0	0.0
0.61-0.70	5	1	20.0	2	40.0	1	20.0	1	20.0
0.71-0.80	4	1	25.0	1	25.0	1	25.0	1	25.0
0.81-0.90	4	0	0.0	1	25.0	1	25.0	2	50.0
0.91-1.00	12	0	0.0	2	16.7	3	25.0	7	58.3
**Total**	285	181	N.A.	80	N.A.	10	N.A.	14	N.A.

Model Coefficient	AS		Grade 0		Pre 1A/B		Pre 2-3A		Grade 1A/B		Grade 2-3A	
	n	%	n	%	n	%	n	%	n	%	n	%
0.00-0.10	16	8.8	82	45.3	34	18.8	3	1.7	46	25.4	0	0.0
0.11-0.20	1	4.0	10	40.0	5	20.0	2	8.0	7	28.0	0	0.0
0.21-0.30	0	0.0	10	40.0	5	20.0	1	4.0	8	32.0	1	4.0
0.31-0.40	0	0.0	4	40.0	0	0.0	0	0.0	4	40.0	2	20.0
0.41-0.50	0	0.0	2	15.4	2	15.4	0	0.0	6	46.2	3	23.1
0.51-0.60	0	0.0	1	16.7	1	16.7	0	0.0	3	50.0	1	16.7
0.61-0.70	0	0.0	0	0.0	1	20.0	0	0.0	2	40.0	2	40.0
0.71-0.80	0	0.0	0	0.0	0	0.0	1	25.0	1	25.0	2	50.0
0.81-0.90	0	0.0	0	0.0	0	0.0	0	0.0	1	25.0	3	75.0
0.91-1.00	0	0.0	0	0.0	0	0.0	0	0.0	2	16.7	10	83.3
**Total**	17	N.A.	109	N.A.	48	N.A.	7	N.A.	80	N.A.	24	N.A.

Data were obtained by flow cytometry after EV capture using membrane sensing peptides. The number (n) and proportion (%) of patients stratified for outcome (rejection episodes) is shown according to the rRF prediction model. Patients were stratified according to rejection grade (higher part of the table; ACR grade 0 *vs.* grade 1A/B *vs.* grade 2 *vs.* grade 3A) or according to the time-point of evaluation (lower part of the table): after surgery (first sampling after heart transplant); grade 0 (non-rejecting patients); ACR grade 1A/B; pre 1A/B (time-point of evaluation before a diagnosis of 1A/B ACR); ACR grade 2-3A; pre 2-3A (time-point of evaluation before a diagnosis of 2-3A ACR). N.A., Not Applicable.

Table S14 – EV surface profiling after MSP-capturing in an independent validation cohort

EV antigen	**Grade 0** (n=32)			**Grade 1A/B** (n=16)			**Grade 2** (n=5)			**Grade 3A** (n=4)			***P*-value**
	25th	*50th*	*75th*	*25th*	*50th*	*75th*	*25th*	*50th*	*75th*	*25th*	*50th*	*75th*	
**Tetrasp.**	3.7	6.9	12.3	14.7	20.5	22.7	17.2	17.6	22.8	28.3	33.9	38.2	**<0.001**
**CD2**	4.0	5.8	11.3	9.4	14.5	19.6	17.0	21.6	30.6	16.8	26.4	31.5	**<0.001**
**CD3**	3.9	5.7	15.0	11.6	13.6	18.2	12.9	15.3	28.0	12.0	17.3	32.0	**0.002**
**CD4**	3.8	7.1	13.6	13.7	15.8	22.7	18.1	21.4	26.0	19.0	25.2	35.2	**<0.001**
**CD8**	5.3	10.8	17.3	13.5	20.2	36.2	22.4	36.6	49.4	13.9	30.4	86.3	**0.002**
**CD19**	5.6	8.8	20.9	15.8	25.8	31.1	23.2	31.9	75.4	15.0	24.7	59.9	**0.001**
**CD20**	9.3	14.6	22.0	26.2	35.0	43.9	12.3	25.1	75.3	31.8	42.2	65.3	**<0.001**
**CD24**	8.4	12.4	16.8	19.8	24.4	38.5	22.6	54.1	78.7	25.7	44.9	79.7	**<0.001**
**CD25**	5.1	7.8	11.9	9.8	17.0	27.9	13.8	26.7	37.9	6.4	20.6	36.5	**0.003**
**CD45**	5.0	12.0	26.0	15.4	26.4	44.3	11.8	28.4	64.5	31.1	50.0	51.8	**0.004**
**CD49e**	9.2	15.4	28.4	24.7	44.9	64.2	40.6	47.2	62.9	12.8	56.0	135.4	**<0.001**
**CD62p**	11.3	13.6	28.1	28.1	36.8	41.7	28.3	41.4	85.7	51.7	58.3	62.9	**<0.001**
**CD142**	14.4	18.6	40.9	39.8	56.4	65.1	73.4	79.5	88.4	51.7	64.6	112.5	**<0.001**
**CD209**	2.6	4.1	9.1	7.4	11.6	16.0	15.9	18.9	22.2	8.6	13.7	29.9	**<0.001**
**HLA-I**	19.6	29.5	48.4	43.6	69.0	77.5	50.1	65.5	78.0	64.1	101.3	170.6	**<0.001**

EV surface antigen profiling by flow cytometry after membrane sensing peptide (MSP)- capturing in an independent validation cohort (n=5; 57 analyzed samples). Median fluorescence intensity (MFI; expressed as arbitrary unit, a.u.) is reported for tetraspanins (CD9, CD63, and CD81) and for a customized panel of 14 EV markers differentially expressed in rejecting recipients (see also Supplementary Data S2). Patients were stratified according to rejection grade (from 0 to 3A). Data are expressed as median and interquartile range; a P-value less than 0.05 was considered significant and reported in bold.

Table S15 – EV surface profiling after immuno-capturing in antibody-mediated rejection

EV antigen	**Grade 0** (n=13)			**AMR** (n=13)			*P*-value
	25th	*50th*	*75th*	*25th*	*50th*	*75th*	
**Tetrasp.**	7.9	15.2	24.0	28.8	42.4	54.1	**0.004**
**CD2**	0.7	2.0	3.7	1.7	6.3	15.9	**0.033**
**CD3**	1.4	6.4	13.4	5.3	9.1	21.8	0.107
**CD4**	1.8	5.4	11.8	1.5	7.9	14.9	0.308
**CD8**	13.1	18.7	23.1	19.6	29.9	45.9	**0.026**
**CD19**	3.2	9.3	13.2	4.8	18.9	37.7	**0.014**
**CD20**	1.9	10.2	18.9	3.2	14.4	25.5	**0.041**
**CD24**	2.6	7.6	11.6	4.6	10.0	16.2	0.079
**CD25**	1.7	3.6	6.7	4.7	9.0	16.4	**0.003**
**CD45**	3.1	8.9	15.1	5.2	13.9	19.8	**0.012**
**CD49e**	7.1	10.9	13.1	6.7	11.9	17.4	0.311
**CD62p**	36.5	72.7	116.5	44.1	81.1	127.1	0.533
**CD142**	1.2	5.1	12.2	2.6	7.3	13.7	0.526
**CD209**	2.5	5.3	9.5	2.9	6.7	10.9	0.607
**HLA-I**	5.4	14.6	22.4	22.2	34.8	56.6	**0.007**

EV surface antigen profiling by flow cytometry after immuno-capturing in patients with antibody-mediated rejection (AMR), as compared to non-rejecting patients (grade 0; matched samples from the same patient; re-analysis of data from Castellani et al.[10]). Median fluorescence intensity (MFI; expressed as arbitrary unit, a.u.) is reported for tetraspanins (CD9, CD63, and CD81) and for a customized panel of 14 EV markers differentially expressed in rejecting recipients (see also Supplementary Data S2). Data are expressed as median and interquartile range; a *P*-value less than 0.05 was considered significant and reported in bold.

Table S16 – Likelihood of rejection according to AI and EV profiling after immuno-capturing

Model Coefficient	Total	Grade 0		Grade 1A/B		Grade 2		Grade 3A	
		n	%	n	%	n	%	n	%
0.00-0.10	144	110	76.4	34	23.6	0	0.0	0	0.0
0.11-0.20	42	30	71.4	12	28.6	0	0.0	0	0.0
0.21-0.30	32	23	71.9	6	18.8	2	6.3	1	3.1
0.31-0.40	8	4	50.0	1	12.5	2	25.0	1	12.5
0.41-0.50	12	7	58.3	2	16.7	2	16.7	1	8.3
0.51-0.60	13	3	23.1	9	69.2	0	0.0	1	7.7
0.61-0.70	11	2	18.2	8	72.7	0	0.0	1	9.1
0.71-0.80	4	0	0.0	3	75.0	0	0.0	1	25.0
0.81-0.90	7	0	0.0	4	57.1	1	14.3	2	28.6
0.91-1.00	12	2	16.7	1	8.3	3	25.0	6	50.0
**Total**	285	181	N.A.	80	N.A.	10	N.A.	14	N.A.

Model Coefficient	AS		Grade 0		Pre 1A/B		Pre 2-3A		Grade 1A/B		Grade 2-3A	
	n	%	n	%	n	%	n	%	n	%	n	%
0.00-0.10	3	2.1	76	52.8	29	20.1	2	1.4	34	23.6	0	0.0
0.11-0.20	10	23.8	14	33.3	6	14.3	0	0.0	12	28.6	0	0.0
0.21-0.30	4	12.5	11	34.4	7	21.9	1	3.1	6	18.8	3	9.4
0.31-0.40	0	0.0	2	25.0	1	12.5	1	12.5	1	12.5	3	37.5
0.41-0.50	0	0.0	3	25.0	2	16.7	2	16.7	2	16.7	3	25.0
0.51-0.60	0	0.0	3	23.1	0	0.0	0	0.0	9	69.2	1	7.7
0.61-0.70	0	0.0	0	0.0	2	18.2	0	0.0	8	72.7	1	9.1
0.71-0.80	0	0.0	0	0.0	0	0.0	0	0.0	3	75.0	1	25.0
0.81-0.90	0	0.0	0	0.0	0	0.0	0	0.0	4	57.1	3	42.9
0.91-1.00	0	0.0	0	0.0	1	8.3	1	8.3	1	8.3	9	75.0
**Total**	17	N.A.	109	N.A.	48	N.A.	7	N.A.	80	N.A.	24	N.A.

Data were obtained by flow cytometric analysis after EV immuno-capturing. The number (n) and proportion (%) of patients stratified for outcome (rejection episodes) is shown according to the rRF prediction model. Patients were stratified according to rejection grade (higher part of the table; ACR grade 0 *vs.* grade 1A/B *vs.* grade 2 *vs.* grade 3A) or according to the time-point of evaluation (lower part of the table): after surgery (first sampling after heart transplant); grade 0 (non-rejecting patients); ACR grade 1A/B; pre 1A/B (time-point of evaluation before a diagnosis of 1A/B ACR); ACR grade 2-3A; pre 2-3A (time-point of evaluation before a diagnosis of 2-3A ACR). N.A., Not Applicable.

Table S17 – Bland-Altman analysis of EV profiling after MSP- *vs.* immuno-capturing

EV antigen	Levels by MSP-capturing *minus* immuno-capturing *[Z-Score of the difference]*			
	Median	*SD*	*Lower Limit*	*Upper Limit*
**Tetrasp.**	+3.0	66.5	-127.4	+133.4
**CD2**	+9.1	71.9	-131.9	+149.9
**CD3**	+24.2	83.0	-138.6	+186.9
**CD4**	+7.3	68.8	-127.5	+142.1
**CD8**	+3.8	53.7	-101.5	+109.0
**CD19**	+11.1	70.1	-126.3	+148.6
**CD20**	+4.8	55.5	-104.0	+113.6
**CD24**	+5.0	68.4	-129.0	+139.0
**CD25**	-12.7	127.6	-262.8	+237.4
**CD45**	-5.2	50.6	-104.3	+93.9
**CD49e**	+5.0	90.6	-172.5	+182.5
**CD62p**	+10.4	59.4	-105.9	+126.7
**CD142**	+7.6	67.3	-124.3	+139.5
**CD209**	+13.1	93.1	-169.4	+195.6
**HLA-I**	+7.0	90.4	-170.3	+184.3
**Mean**	**+6.2**	**74.5**	**-139.8**	**+152.2**

The table reports percentage differences of Z-score (median, standard deviation, and lower/upper limit of the 95% confidence interval) for EV levels measured after MSP-capturing *minus* those measured after immuno-capturing (Bland-Altman analysis), for the customized panel of 14 EV markers differentially expressed in rejecting recipients (see also Supplementary Data S2). A positive difference indicates an overestimate of MSP-capturing on immuno-capturing approach, a negative difference an underestimate.

Table S18 – Diagnostic performance of AI model and EV profiling compared to literature

Test	Reference	Study Cohort (ACR)	AUC	*P*-value
AlloMap	Crespo-Leiro et al. 2016	480 (46)	0.700	**<0.001*<br>0.011****
dd-cf DNA	Agbor-Enoh et al. 2021	1072 (49)	0.890	0.063*<br>0.269**
EV profile (MSP-capture)	Burrello et al.*	285 (24)	0.968	N.A.
EV profile (immuno-capture)	Burrello et al.**	285 (24)	0.854	N.A.

The table reports accuracy in the diagnosis of acute cellular rejection (ACR) of the AI model based on EV profiling from the present study as compared to that of AlloMap and cell-free DNA (dd-cf DNA), reported in literature (Crespo-Leiro et al. 2016[11] and Agbor-Enoh et al. 2021[12]). Numerosity of the cohort, number of ACR (grade 2-3A) cases, and AUC (area under the curve) are reported together with their comparison (**P*-value of the comparison with EV profile after MSP-capture; ** *P*-value of the comparison with EV profile after immuno-capture). *P*-value less than 0.05 was considered significant and highlighted in bold.

Supplementary References

1. S. Stewart, G. L. Winters, M. C. Fishbein, et al, Revision of the 1990 working formulation for the standardization of nomenclature in the diagnosis of heart rejection. *J Heart Lung Transplant* **24**, 1710–1720 (2005).
2. G. J. Berry, M. M. Burke, C. Andersen, et al. The 2013 International Society for Heart and Lung Transplantation Working Formulation for the standardization of nomenclature in the pathologic diagnosis of antibody-mediated rejection in heart transplantation. *J Heart Lung Transplant* **32**, 1147–1162 (2013).
3. Peled Y, Lavee J, Ram E, et al. Recurrent acute cellular rejection graded ISHLT 1R early after heart transplantation negatively affects long-term outcomes: The prognostic significance of 1990 ISHLT grades 1B and 2. Transpl Immunol **55**, 101204 (2019).
4. Velleca A, Shullo MA, Dhital K, et al. The International Society for Heart and Lung Transplantation (ISHLT) guidelines for the care of heart transplant recipients. *J Heart Lung Transplant* **42**, e1-e141 (2023).
5. J. Burrello, A. Burrello, E. Vacchi, et al. Supervised and unsupervised learning to define the cardiovascular risk of patients according to an extracellular vesicle molecular signature. *Transl Res* **244**, 114–125 (2022).
6. N. Koliha, Y. Wiencek, U. Heider, et al. A novel multiplex bead-based platform highlights the diversity of extracellular vesicles. *J Extracell Vesicles* **5**, 29975 (2016).
7. O. P. B. Wiklander, R. B. Bostancioglu, J. A. Welsh, et al. Systematic Methodological Evaluation of a Multiplex Bead-Based Flow Cytometry Assay for Detection of Extracellular Vesicle Surface Signatures. *Front Immunol* **9**, 1326 (2018).
8. A. Gori, A. Romanato, B. Greta, et al. Membrane-binding peptides for extracellular vesicles on-chip analysis. *J Extracell Vesicles* **9**, 1751428 (2020).
9. A. Strada, R. Frigerio, G. Bergamaschi, et al. Membrane-Sensing Peptides for Extracellular Vesicle Analysis. *Methods Mol Biol* **2578**, 249–257 (2023).
10. C. Castellani, J. Burrello, M. Fedrigo, et al. Circulating Extracellular Vesicles as a Noninvasive Biomarker of Rejection in Heart Transplant. *J Heart Lung Transplant* **39**, 1136–1148 (2020).
11. Crespo-Leiro MG, Stypmann J, Schulz U, et al. Clinical usefulness of gene-expression profile to rule out acute rejection after heart transplantation: CARGO II. *Eur Heart J* **37**, 2591–2601 (2016).
12. Agbor-Enoh S, Shah P, Tunc I, et al; GRAfT Investigators. Cell-Free DNA to Detect Heart Allograft Acute Rejection. *Circulation* **143**, 1184–1197 (2021).
