# Supplementary material for: Machine learning-assisted assessment of extracellular vesicles can monitor cellular rejection after heart transplant
Source: Commun Med (Lond). 2025 Jul 11;5:288. doi: 10.1038/s43856-025-00999-0 (PMC12246144; doi:10.1038/s43856-025-00999-0)
Supplement: Supplementary file 2 — Description of Additional Supplementary Files [file 43856_2025_999_MOESM2_ESM.pdf]

Description of Additional Supplementary Files

File name- Supplementary Data S1

File description – Clinical-biochemical parameters and endomyocardial biopsy

File name- Supplementary Data S2

File description - EV surface profiling after immuno-capturing

File name- Supplementary Data S3

File description - EV surface profiling after immuno-capturing according to time

File name- Supplementary Data S4

File description - Training and Tuning of an AI model to predict rejection episode
