# Supplementary material for: Machine learning-assisted assessment of extracellular vesicles can monitor cellular rejection after heart transplant
Source: Commun Med (Lond). 2025 Jul 11;5:288. doi: 10.1038/s43856-025-00999-0 (PMC12246144; doi:10.1038/s43856-025-00999-0)
Supplement: Supplementary file 7 — Reporting summary [file 43856_2025_999_MOESM7_ESM.pdf]

nature portfolio

Corresponding author(s): COMMSMED-24-0644-C

Last updated by author(s): 17 May 2025

Reporting Summary

Nature Portfolio wishes to improve the reproducibility of the work that we publish. This form provides structure for consistency and transparency in reporting. For further information on Nature Portfolio policies, see our Editorial Policies and the Editorial Policy Checklist.

Statistics

For all statistical analyses, confirm that the following items are present in the figure legend, table legend, main text, or Methods section.

n/a	Confirmed	
☐	☒	The exact sample size (*n*) for each experimental group/condition, given as a discrete number and unit of measurement
☐	☒	A statement on whether measurements were taken from distinct samples or whether the same sample was measured repeatedly
☐	☒	The statistical test(s) used AND whether they are one- or two-sided<br>*Only common tests should be described solely by name; describe more complex techniques in the Methods section.*
☐	☒	A description of all covariates tested
☐	☒	A description of any assumptions or corrections, such as tests of normality and adjustment for multiple comparisons
☐	☒	A full description of the statistical parameters including central tendency (e.g. means) or other basic estimates (e.g. regression coefficient) AND variation (e.g. standard deviation) or associated estimates of uncertainty (e.g. confidence intervals)
☐	☒	For null hypothesis testing, the test statistic (e.g. *F*, *t*, *r*) with confidence intervals, effect sizes, degrees of freedom and *P* value noted<br>*Give P values as exact values whenever suitable.*
☒	☐	For Bayesian analysis, information on the choice of priors and Markov chain Monte Carlo settings
☒	☐	For hierarchical and complex designs, identification of the appropriate level for tests and full reporting of outcomes
☐	☒	Estimates of effect sizes (e.g. Cohen's *d*, Pearson's *r*), indicating how they were calculated

Our web collection on statistics for biologists contains articles on many of the points above.

Software and code

Policy information about availability of computer code

Data collection	no software was used
Data analysis	IBM SPSS Statistics 26 (IBM Corp, Armonk, NY) and GraphPad Prism 9.0 (GraphPad, La Jolla, CA) were used for descriptive statistics. rRF algorithm was built using a supervised learning method with Python 3.8.10 (library, scikit learn 1.3.1).

For manuscripts utilizing custom algorithms or software that are central to the research but not yet described in published literature, software must be made available to editors and reviewers. We strongly encourage code deposition in a community repository (e.g. GitHub). See the Nature Portfolio guidelines for submitting code & software for further information.

Data

Policy information about availability of data

All manuscripts must include a data availability statement. This statement should provide the following information, where applicable:

- Accession codes, unique identifiers, or web links for publicly available datasets
- A description of any restrictions on data availability
- For clinical datasets or third party data, please ensure that the statement adheres to our policy

ll data needed to evaluate the conclusions in the paper are present 392 in the paper and/or the Supplementary Materials. Additional information may be obtained from corresponding authors upon reasonable request.

Human research participants

Policy information about studies involving human research participants and Sex and Gender in Research.

Reporting on sex and gender	This is a prospective study. All consecutive patients during a one-year inclusion period were enrolled. No specific design was made for gender dimension.
Population characteristics	Mean age was 58 years, 20 males (83.3%) and 4 females. All patients' information are detailed in Table 1 and 2
Recruitment	Consecutive patients undergoing heart transplant between August 2020 and August 2021 were recruited and longitudinally evaluated for the first year after transplant. A total of 24 patients were included in the analysis, with 9-17 visits each (visit median interval of 28 days); at each visit patient underwent clinical evaluation, routine biochemical exams, endomyocardial biopsy (EMB)and blood sampling (blood was collected immediately before biopsy, thus avoiding confoundings related to the procedure).
Ethics oversight	Study protocol (#0062556) was approved by local ethical committee and fully informed written consent was provided by each participant.

Note that full information on the approval of the study protocol must also be provided in the manuscript.

Field-specific reporting

Please select the one below that is the best fit for your research. If you are not sure, read the appropriate sections before making your selection.

☒ Life sciences ☐ Behavioural & social sciences ☐ Ecological, evolutionary & environmental sciences

For a reference copy of the document with all sections, see nature.com/documents/nr-reporting-summary-flat.pdf

Life sciences study design

All studies must disclose on these points even when the disclosure is negative.

Sample size	Consecutive patients undergoing heart transplants between August 2020 and August 2021 were recruited and longitudinally evaluated for the first year after the transplant. A total of 285 samples were collected. The sample size was not determined a priori.
Data exclusions	Antibody mediated rejection (AMR) was detected in two samples (patient #20), which were excluded from further analysis. The analysis was performed to assess assay's sensitivity and specificity in cellular rejection (ACR)
Replication	N/A
Randomization	N/A
Blinding	The investigators who conducted experimental analysis were blind to patients' diagnosis

Reporting for specific materials, systems and methods

We require information from authors about some types of materials, experimental systems and methods used in many studies. Here, indicate whether each material, system or method listed is relevant to your study. If you are not sure if a list item applies to your research, read the appropriate section before selecting a response.

Materials & experimental systems

n/a	Involved in the study	
☐	☒	Antibodies
☒	☐	Eukaryotic cell lines
☒	☐	Palaeontology and archaeology
☒	☐	Animals and other organisms
☐	☒	Clinical data
☒	☐	Dual use research of concern

Methods

n/a	Involved in the study	
☒	☐	ChIP-seq
☐	☒	Flow cytometry
☒	☐	MRI-based neuroimaging

Antibodies

Antibodies used	37 antigens was evaluated by a standardized commercially available kit (Figure 2A; MACSPlex Human Exosome Kit; Miltenyi Biotec, Bergisch Gladbach, Germany). Moreover the following antibodies were used CD2-PE-Dazzle 594, CD4-BV421, CD8-PC7, CD9-BV510, CD19-BV650, CD25-AF700, CD49e-BV750, CD63-BV605, CD209-FITC, HLA-ABC APC (BioLegend, USA); CD3-BUV496, CD45-BUV615, CD62p-BUV805, CD81-BUV737 (BD Bioscience, USA); CD24-PE, CD142-PerCP Vio700 (Miltenyi Biotech, Germany); CD20-APC AF750 (Beckman&Coulter, USA).
Validation	The AI model was validated by a leave-one-out algorithm, which randomly selects N-1 patients, trains the rRF into this cohort and tests the trained model on the remaining subject; the process is reiterated N times (where N is the number of patients included in the analysis), with the test subject rotating at each round. Model accuracy at validation results from the mean of accuracy obtained at each round on the test patient.

Clinical data

Policy information about clinical studies

All manuscripts should comply with the ICMJE guidelines for publication of clinical research and a completed CONSORT checklist must be included with all submissions.

Clinical trial registration	N/A
Study protocol	#0062556
Data collection	From August 2020, 24 consecutive patients, which underwent heart transplant in the same referral centre, according to the same protocols, was enrolled and longitudinally followed with scheduled visits for a median of 303 days
Outcomes	Diagnosis and grading (from R0-to-R3) of ACR were defined according to guidelines of the International Society for heart and Lung Transplantation.

Flow Cytometry

Plots

Confirm that:

☒ The axis labels state the marker and fluorochrome used (e.g. CD4-FITC).

☒ The axis scales are clearly visible. Include numbers along axes only for bottom left plot of group (a 'group' is an analysis of identical markers).

☒ All plots are contour plots with outliers or pseudocolor plots.

☒ A numerical value for number of cells or percentage (with statistics) is provided.

Methodology

Sample preparation	Blood was drawn into tubes containing EDTA and centrifuged at 1,600g for 15 minutes to precipitate cellular components; low-centrifuge speed was used to prevent platelet activation due to shearstress–induced. Free-platelet plasma underwent serial centrifugation cycles to remove cellular debris and larger EVs: 3,000g 20min, 10,000g 15min, and 20,000g 30min at 4° C. All samples underwent to systematic profiling of EV surface antigens according to two different protocols.
Instrument	Symphony A5 (BD Bioscience). MACSQuant Analyzer 10 flow cytometer (Miltenyi Biotec; Bergisch Gladbach, Germany).
Software	FlowJo™ Software; iMACSQuantify™ Software
Cell population abundance	At least 10'000 single-bead events were collected and further analyzed based on the median fluorescence in each specific channel
Gating strategy	Median fluorescence intensity (MFI) was measured for each subset of capture beads, corrected by subtracting the signal of corresponding blank controls, and normalized by the mean MFI of CD9, CD63, and CD81 (normalized MFI, nMFI). For the peptide -based analysis . All the antibodies were used at a final concentration of 0.5 ug/mL, adjusting the staining volume to 100 uL with PBS supplemented with 0.5 mg/mL of human IgG and 0.5% human albumin, to reduce unspecific binding. In the second tube, we used the membrane dye Memglow700 (Cytoskeleton, USA) to normalize the membrane signal; MemGlow was used at a final concentration of 50 nM in 100 uL PBS. After staining, samples were magnetically separated to remove all the unbound dyes, resuspended in 100 uL of PBS, and acquired by Symphony A5 (BD Bioscience). At least 10'000 single-bead events were collected and further analyzed based on the median fluorescence in each specific channel.

☐ Tick this box to confirm that a figure exemplifying the gating strategy is provided in the Supplementary Information.
